# Supplementary material for: Mechanism of signal-anchor triage during early steps of membrane protein insertion
Source: Mol Cell. 2023 Mar 16;83(6):961–973.e7. doi: 10.1016/j.molcel.2023.01.018 (PMC10155758; doi:10.1016/j.molcel.2023.01.018)
Supplement: Document S1. Figures S1–S6 and supplemental references [file mmc1.pdf]

Molecular Cell, Volume *83*

Supplemental information

Mechanism of signal-anchor triage

during early steps of membrane protein insertion

Haoxi Wu and Ramanujan S. Hegde

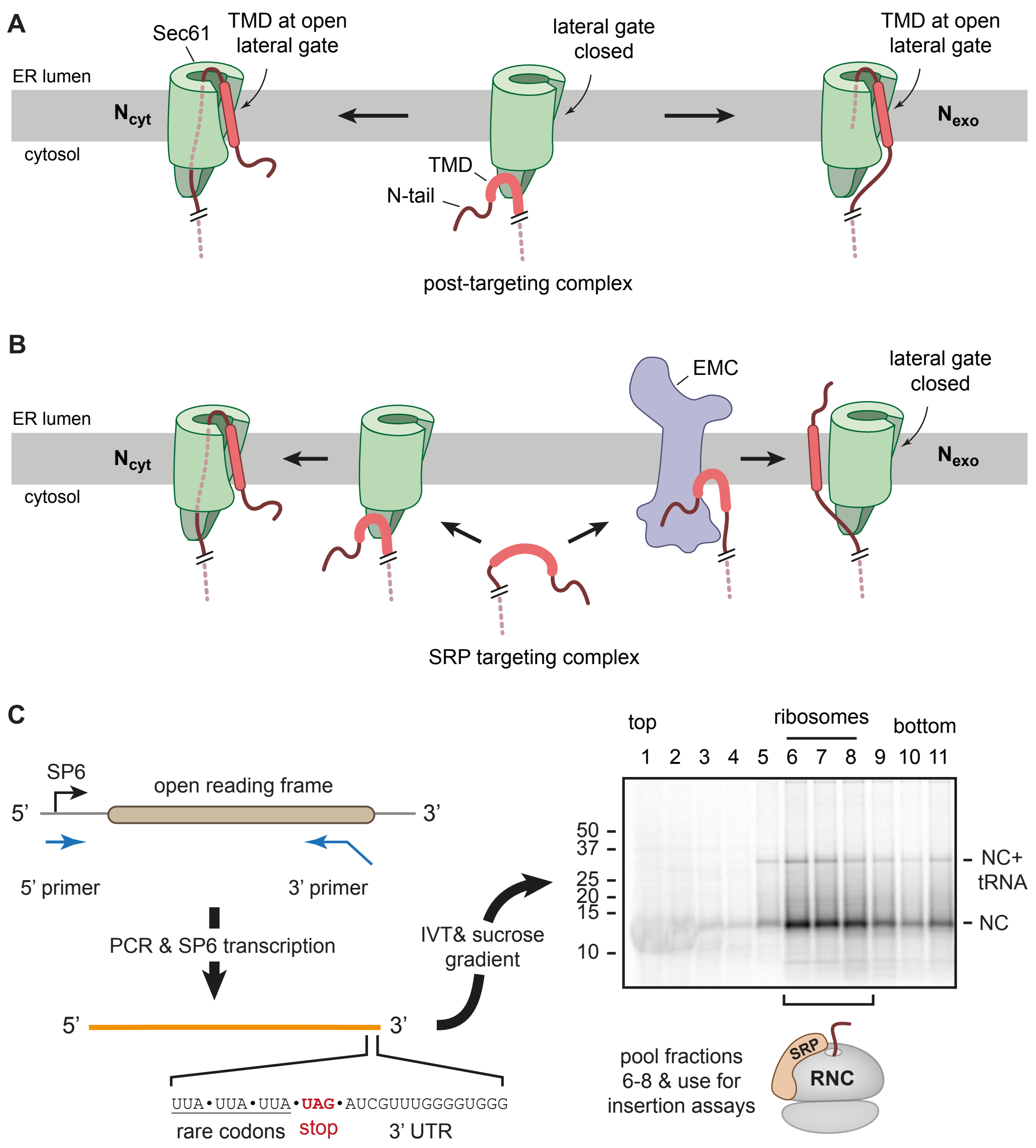

Figure S1. Models of membrane protein topogenesis, related to Figure 1

(A) Model for Sec61-mediated SA topogenesis based on Higy et al., 2004 [S1]. After targeting to the membrane, RNCs dock at the Sec61 complex. The SA is inserted in either the N_{exo} or N_{cyt} orientation. In both cases, the SA passes through Sec61's lateral gate, with one of the SA's flanking domains translocating through Sec61's central channel.

(B) Model for SA topogenesis based on Chitwood and Hegde, 2019 [S2]. After targeting to the membrane, N_{exo} insertion is mediated by the EMC insertase, after which the ribosome docks at the Sec61 complex. N_{cyt} insertion is mediated by the Sec61 complex.

(C) Method to produce RNCs stalled at a defined site by in vitro translation. The template for transcription is generated by PCR with a 3' primer that encodes three consecutive rare codons (TTA), a stop codon and a 3'UTR sequence. The PCR product is transcribed in vitro and translated in RRL containing only its endogenous tRNAs. RRL contains limited amounts of the tRNA used to decode the UUA codon [S3, S4], causing stalling at the three UUA codons. The translation reaction is separated by centrifugation through a 10-50% sucrose gradient and the RNCs are collected for downstream assays. A representative example (TAAR5-SA+70) is shown on the right after SDS-PAGE and autoradiography. Intact RNCs co-fractionate with ribosomes in fractions 6-8. Note that the peptidyl-tRNA bond on most nascent chains is hydrolyzed during electrophoresis at basic pH, but is stable at neutral pH.

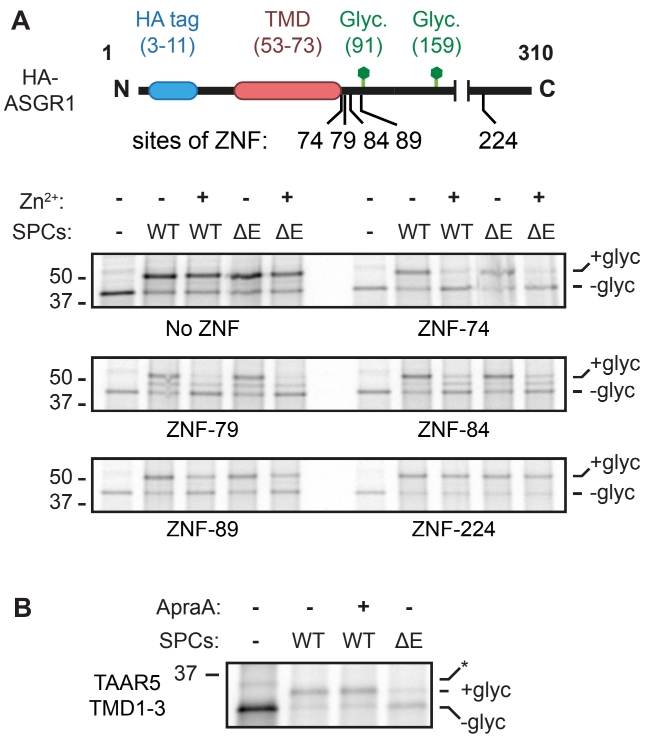

Figure S2. EMC-dependent SA insertion requires late targeting, related to Figure 2

(A) Top: diagram of HA-ASGR1 constructs without or with a zinc finger from ADR1 (ZNF) inserted immediately at the indicated positions. Bottom: ^{35}S-methionine labeled HA-ASGR1 constructs lacking or containing ZNF were translated in the absence or presence of SPCs from wild type (WT) or ΔEMC (ΔE) HEK293 cells without or with Zn^{2+} where indicated. After translation, the SPCs were recovered by centrifugation and analyzed by SDS-PAGE and autoradiography. An aliquot of the sample lacking SPCs was analyzed directly. The glycosylated (+glyc.) and non-glycosylated (-glyc.) products are indicated. Panels of ZNF, ZNF-74, ZNF-224 are reproduced from Fig. 2A for comparison.

(B) TAAR5(TMD1-3) is EMC-dependent. ^{35}S-methionine labeled TAAR5(TMD1-3) was translated in the absence or presence of SPCs derived from wild type (WT) or ΔEMC (ΔE) HEK293 cells. The Sec61 lateral gate inhibitor ApraA was included where indicated. After translation, the SPCs were recovered by centrifugation and analyzed by SDS-PAGE and autoradiography. An aliquot of the sample lacking SPCs was analyzed directly. The glycosylated (+glyc.) and non-glycosylated (-glyc.) products are indicated. Asterisk indicates a product seen in samples lacking SPCs, and may represent ubiquitin-modified substrate.

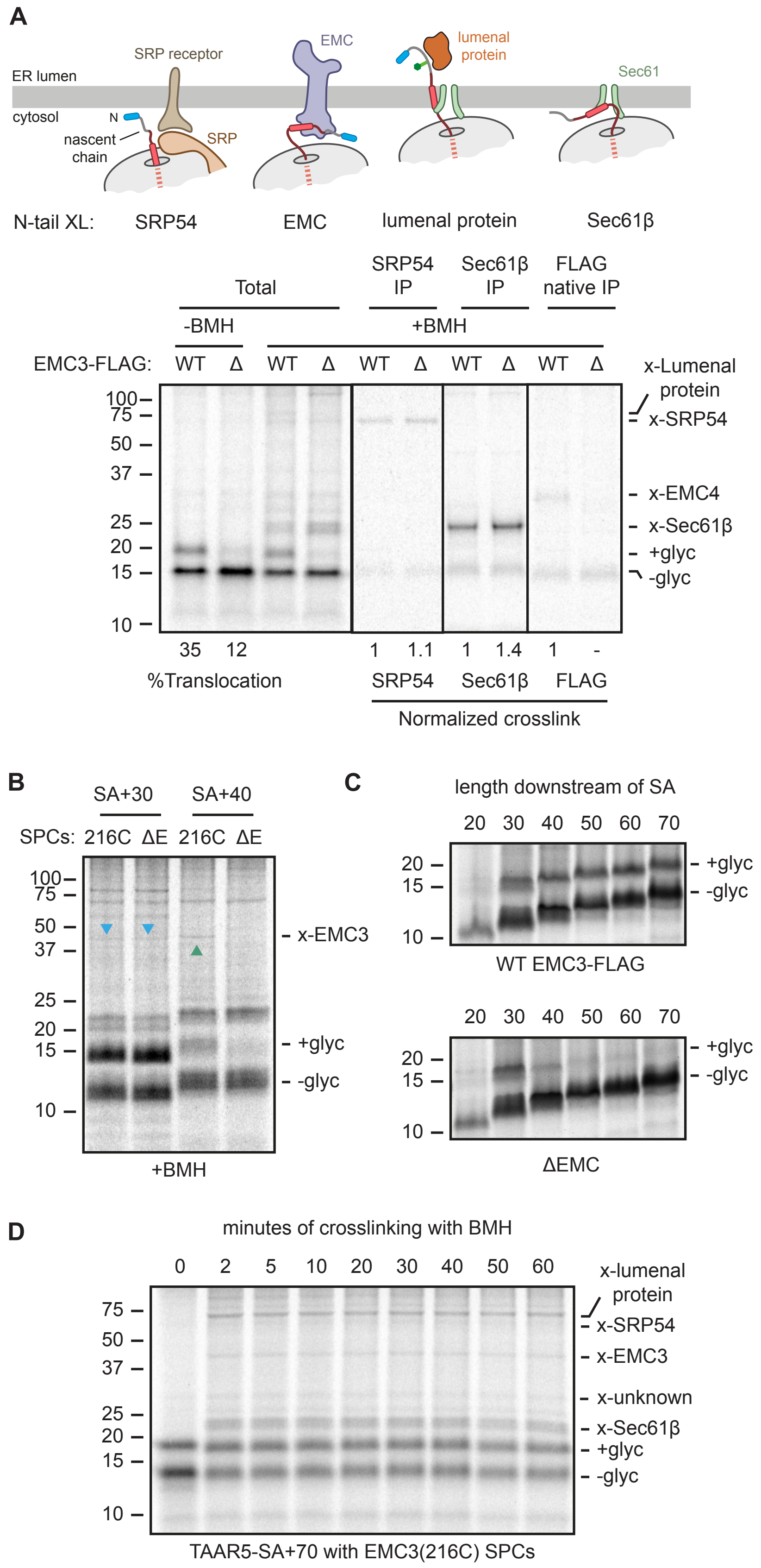
A
ER lumen
cytosol
SRP receptor
N
nascent chain
SRP
EMC
lumenal protein
Sec61
N-tail XL: SRP54
EMC
lumenal protein
Sec61β
Total
SRP54 IP
Sec61β IP
FLAG native IP
-BMH
+BMH
EMC3-FLAG: WT Δ WT Δ WT Δ WT Δ WT Δ
100
75
50
37
25
20
15
10
x-Lumenal protein
x-SRP54
x-EMC4
x-Sec61β
+glyc
-glyc
35 12
%Translocation
1 1.1
1 1.4
1 -
SRP54
Sec61β
FLAG
Normalized crosslink
B
SA+30
SA+40
SPCs: 216C ΔE 216C ΔE
x-EMC3
+glyc
-glyc
+BMH
C
length downstream of SA
20 30 40 50 60 70
+glyc
-glyc
WT EMC3-FLAG
ΔEMC
D
minutes of crosslinking with BMH
0 2 5 10 20 30 40 50 60
x-lumenal protein
x-SRP54
x-EMC3
x-unknown
x-Sec61β
+glyc
-glyc
TAAR5-SA+70 with EMC3(216C) SPCs

Figure S3. Detection of EMC-substrate interaction, related to Figure 3

(A) Top: diagram showing putative factors sampled by the N-tail of an SA-containing RNC. Bottom: ^{35}S-methionine labeled TAAR5-SA+70 RNCs were incubated with SPCs containing EMC3(WT)-FLAG or lacking EMC (ΔE). One aliquot was analyzed directly (-BMH) and another treated with BMH, a sulfhydryl-reactive homo-bifunctional crosslinker (+BMH). The crosslinked products were analyzed directly, after denaturing IP with indicated antibodies or after native IP of EMC3 via the FLAG tag. The non-glycosylated (-glyc) and glycosylated (+glyc) products and crosslinks to Sec61β, EMC4, SRP54 and an ER lumenal protein are indicated. Prior to SDS-PAGE, tRNA was digested from the nascent chain using RNase A.

(B) Crosslinking analysis of TAAR5-SA+30 and TAAR5-SA+40 RNCs. ^{35}S-methionine labeled RNCs of TAAR5-SA+30 and TAAR5-SA+40 were incubated with SPCs containing EMC3(216C)-FLAG or lacking EMC (ΔE), crosslinked with BMH, and analyzed directly. The EMC3 crosslink is indicated with an upward green arrow. In TAAR5-SA+30 samples, a crosslink product of unknown identity with similar size to EMC3 is marked with downward blue arrows. This product remains in ΔEMC SPCs.

(C) Insertion assays for RNCs of various lengths and their dependence on EMC. ^{35}S-methionine labeled TAAR5 RNCs of indicated length were incubated with WT or ΔEMC (ΔE) SPCs. The glycosylated (+glyc) and non-glycosylated (-glyc) products are indicated. Note that prior to SDS-PAGE, tRNA was digested from the nascent chain using RNase A.

(D) Time course of BMH crosslinking. ^{35}S-methionine labeled TAAR5-SA+70 RNCs were incubated with EMC3(216C)-FLAG SPCs, crosslinked with BMH for the indicated duration, and analyzed directly.

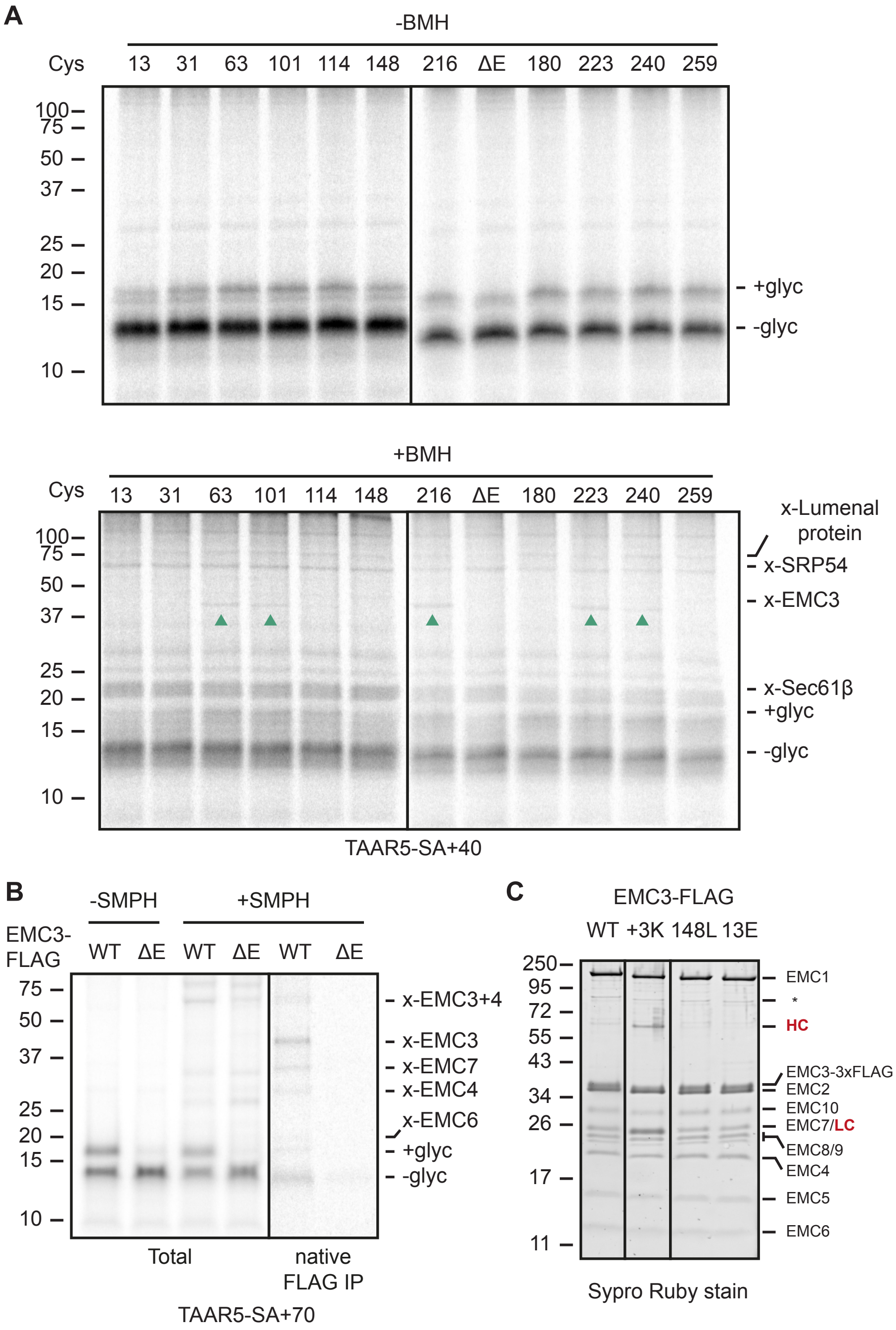

Figure S4. Mapping of EMC-substrate interactions, related to Figure 4

(A) Mapping substrate interaction with single cysteine variants of EMC3. ^{35}S-methionine labeled TAAR5-SA+40 RNCs were incubated with indicated EMC3-FLAG single cysteine variant SPCs, crosslinked with BMH and analyzed directly. The non-glycosylated (-glyc) and glycosylated (+glyc) products and crosslinks to Sec61β, EMC3 (upward green arrows), SRP54 and an ER lumenal protein are indicated. Prior to SDS-PAGE, tRNA was digested from the nascent chain using RNase A.

(B) Crosslinking reactions between ^{35}S-methionine labeled TAAR5-SA+70 RNCs and SPCs stably expressing EMC3-FLAG (WT) versus SPCs lacking EMC (ΔE). Crosslinking was with the sulfhydryl-amine heterobifunctional crosslinker SMPH. Samples were analyzed directly (total) or after native IP of EMC3 via the FLAG tag. The non-glycosylated (-glyc) and glycosylated (+glyc) products and EMC crosslinks consistent with EMC4, EMC6 and EMC7 are indicated.

(C) EMC3 mutations do not affect EMC integrity or abundance. Cells continuously expressing EMC3-FLAG (WT or indicated mutations) were harvested and EMC was affinity purified under native conditions via the FLAG tag. The products eluted from the affinity resin using FLAG peptide were analyzed by SDS-PAGE and Sypro-Ruby staining. Note that all EMC subunits are recovered in identical abundances and ratios for each of the mutants compared to the WT. A small amount of IgG heavy chain (HC) and light chain (LC) contaminate the +3K sample, and are indicated. The samples are all from the same experiment analyzed on the same gel, with vertical lines indicating places where intervening lanes of other samples were removed.

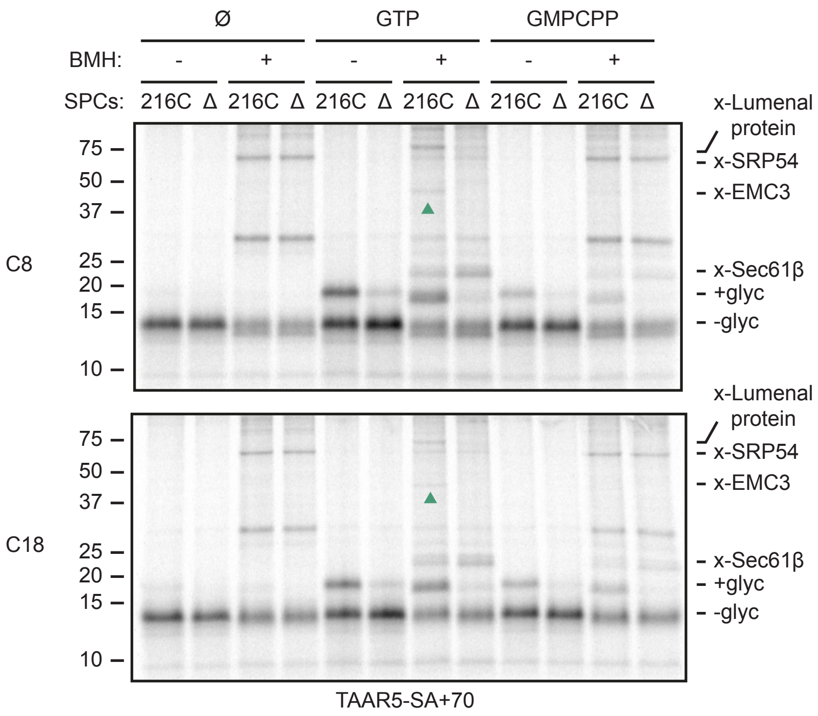

Figure S5. SRP release is required for EMC-substrate interaction, related to Figure 5

^{35}S-methionine labeled TAAR5-SA+70 RNCs with a cysteine at position 8 (top) or 18 (bottom) were mixed with GTP, the slowly-hydrolyzed GTP analog GMPCPP or nothing (Ø), then incubated with SPCs containing EMC3(216C)-FLAG or lacking EMC (Δ). Pelleted SPCs were subject to BMH crosslinking as indicated and analyzed directly. The non-glycosylated (-glyc) and glycosylated (+glyc) products and crosslinks to Sec61β, EMC3 (upward green arrows), SRP54 and a lumenal protein are indicated.

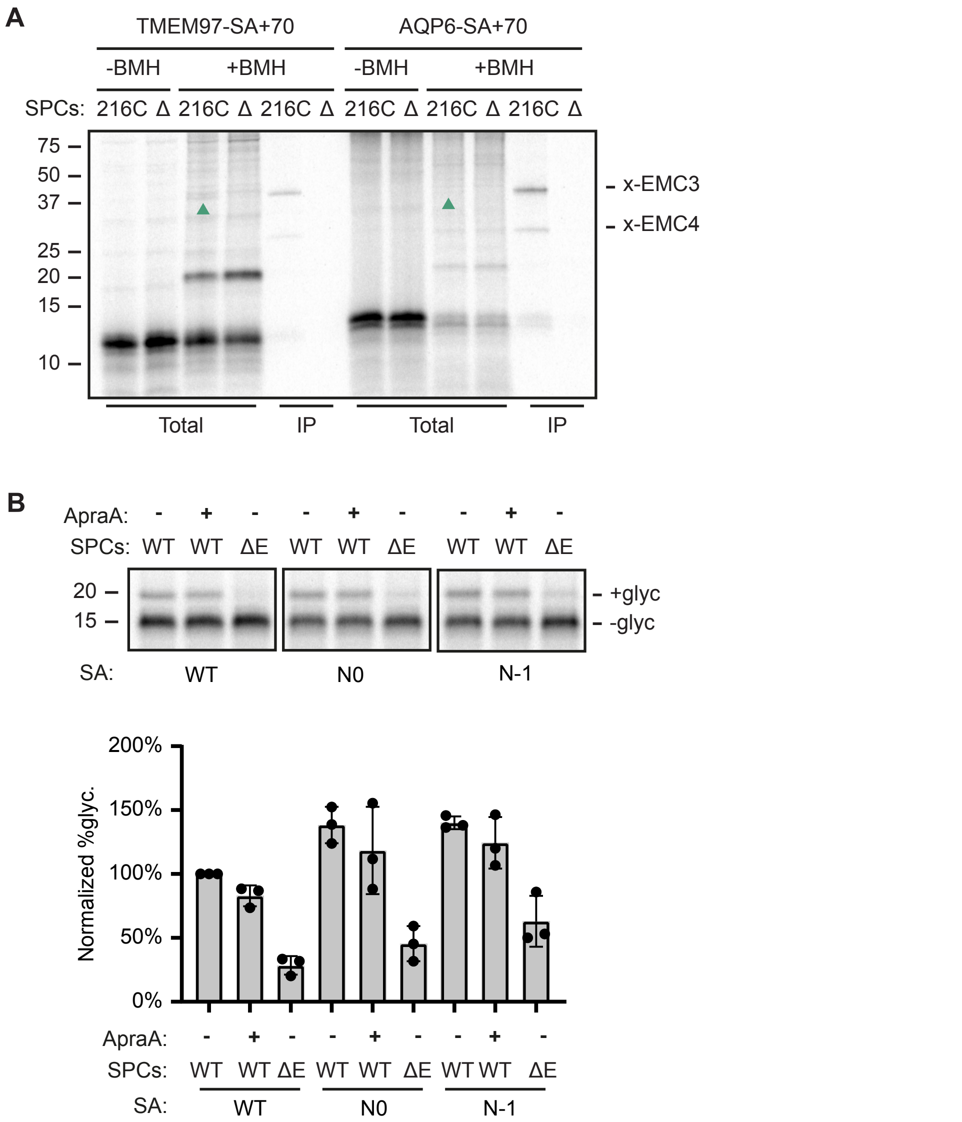

Figure S6. Determinants for EMC-substrate interaction, related to Figure 6

(A) ^{35}S-methionine labeled RNCs of TMEM97-SA+70 or AQP6-SA+70 were incubated with SPCs stably expressing EMC3(216C)-FLAG or lacking EMC (ΔE). One aliquot was analyzed directly (-BMH) and another treated with BMH (+BMH). The crosslinked products were analyzed directly, or after native IP of EMC3 via the FLAG tag. The non-glycosylated (-glyc) and glycosylated (+glyc) products and crosslinks to EMC3 (upward green arrows) and EMC4 are indicated. Prior to SDS-PAGE, tRNA was digested from the nascent chain using RNase A.

(B) ^{35}S-methionine labeled RNCs of the indicated TAAR5-SA+70 variants were incubated with SPCs stably expressing either wild type EMC3-FLAG or lacking EMC (ΔE). ApraA was included where indicated. The SPCs were recovered by centrifugation and analyzed by SDS-PAGE and autoradiography. Quantification of %glycosylation (indicative of insertion in the N_{exo} orientation) from three such experiments is shown at the bottom.

SUPPLEMENTAL REFERENCES

S1. Higy, M., Junne, T., and Spiess, M. (2004). Topogenesis of membrane proteins at the endoplasmic reticulum. Biochemistry *43*, 12716–12722. 10.1021/bi048368m.

S2. Chitwood, P.J., and Hegde, R.S. (2019). The Role of EMC during Membrane Protein Biogenesis. Trends Cell Biol. *29*, 371–384. 10.1016/j.tcb.2019.01.007.

S3. Chandrasekaran, V., Juszkiewicz, S., Choi, J., Puglisi, J.D., Brown, A., Shao, S., Ramakrishnan, V., and Hegde, R.S. (2019). Mechanism of ribosome stalling during translation of a poly(A) tail. Nat. Struct. Mol. Biol. *26*, 1132–1140. 10.1038/s41594-019-0331-x.

S4. Feng, Q., and Shao, S. (2018). In vitro reconstitution of translational arrest pathways. Methods *137*, 20–36. 10.1016/j.ymeth.2017.12.018.
