# Supplementary material for: Mechanism of signal-anchor triage during early steps of membrane protein insertion
Source: Mol Cell. 2023 Mar 16;83(6):961–973.e7. doi: 10.1016/j.molcel.2023.01.018 (PMC10155758; doi:10.1016/j.molcel.2023.01.018)
Supplement: Document S2. Article plus supplemental information [file mmc2.pdf]

Molecular Cell

Article

Mechanism of signal-anchor triage during early steps of membrane protein insertion

Graphical abstract

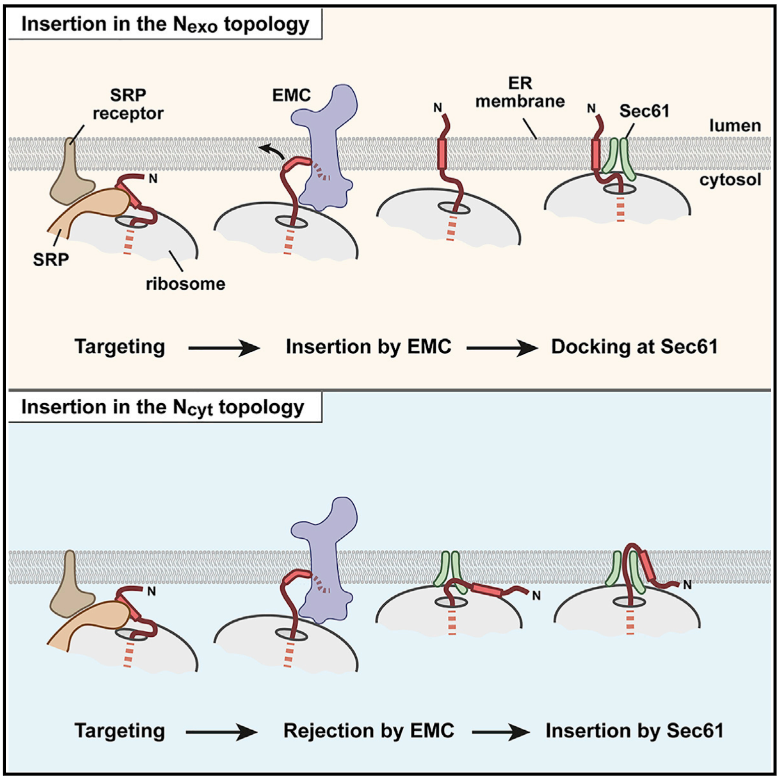

Authors

Haoxi Wu, Ramanujan S. Hegde

Correspondence

rhegde@mrc-lmb.cam.ac.uk

In brief

The first transmembrane domain of most membrane proteins serves as both a targeting signal and a membrane anchor. How such signal anchors are inserted in the correct topology has long been debated. Wu and Hegde now show that signal-anchor topogenesis involves sequential triage by the EMC insertase and Sec61 channel.

Highlights

- Signal anchors can use either EMC or Sec61 for membrane insertion at the ER
- EMC samples signal anchors after SRP release before ribosome docking on Sec61
- A hydrophilic vestibule in EMC houses the N-tail of substrates before translocation
- Signal anchors that are not inserted after sampling EMC can then insert via Sec61

Wu & Hegde, 2023, Molecular Cell *83*, 961–973
March 16, 2023 © 2023 MRC Laboratory of Molecular Biology.
Published by Elsevier Inc.
https://doi.org/10.1016/j.molcel.2023.01.018

Molecular Cell

CellPress
OPEN ACCESS

Article

Mechanism of signal-anchor triage during early steps of membrane protein insertion

Haoxi Wu[1] and Ramanujan S. Hegde[1,2,*]
[1]MRC Laboratory of Molecular Biology, Cambridge CB2 0QH, UK
[2]Lead contact
*Correspondence: rhegde@mrc-lmb.cam.ac.uk
https://doi.org/10.1016/j.molcel.2023.01.018

SUMMARY

Most membrane proteins use their first transmembrane domain, known as a signal anchor (SA), for co-translational targeting to the endoplasmic reticulum (ER) via the signal recognition particle (SRP). The SA then inserts into the membrane using either the Sec61 translocation channel or the ER membrane protein complex (EMC) insertase. How EMC and Sec61 collaborate to ensure SA insertion in the correct topology is not understood. Using site-specific crosslinking, we detect a pre-insertion SA intermediate adjacent to EMC. This intermediate forms after SA release from SRP but before ribosome transfer to Sec61. The polypeptide's N-terminal tail samples a cytosolic vestibule bordered by EMC3, from where it can translocate across the membrane concomitant with SA insertion. The ribosome then docks on Sec61, which has an opportunity to insert those SAs skipped by EMC. These results suggest that EMC acts between SRP and Sec61 to triage SAs for insertion during membrane protein biogenesis.

INTRODUCTION

Approximately 25% of protein-coding genes in most organisms encode integral membrane proteins with diverse functions across biology.[1,2] Nearly all membrane proteins are initially inserted into the endoplasmic reticulum (ER) membrane in eukaryotes or plasma membrane in prokaryotes.[3] The defining feature of a membrane protein is at least one transmembrane domain (TMD). Hence, a critical step in the biogenesis of a membrane protein is the insertion of its TMD(s) into the lipid bilayer in the correct topology (i.e., orientation). Despite decades of study,[4] protein topogenesis remains incompletely understood.[5]

Particularly challenging to understand are membrane proteins whose first TMD serves as a signal sequence for co-translational targeting and are called signal anchors (SAs). Among the ~5,000 human membrane proteins, ~2,600 contain an SA preceded by an N-tail of fewer than 100 aa. Such SAs typically favor the orientation that places flanking positive charges in the cytosol.[6–8] However, most short unstructured N-tails (~2,200) contain low or no charge, which is compatible with SA insertion in either the N_{exo} or N_{cyt} topology (in which the N-tail faces the exoplasmic or cytosolic side of the membrane, respectively). Furthermore, SA length and hydrophobicity also affect topology.[9,10] How the protein translocation machinery interprets these sequence features to determine SA topology continues to be debated.

The prevailing model has been that the Sec61 protein translocation channel inserts SAs of both orientations[11] (Figure S1A). The alpha subunit of the heterotrimeric Sec61 complex forms a membrane-spanning central channel that can also open toward the lipid bilayer via a lateral gate.[12,13] N_{cyt} SAs engage this lateral gate in a hairpin configuration such that the N-tail faces the cytosol and C-tail is pulled into the channel. N_{exo} SAs are postulated to engage the lateral gate in the opposite orientation to facilitate its translocation. Both types of SA have been detected adjacent to Sec61 by photo-crosslinking at early stages of insertion, after which they would diffuse into the membrane to set the protein's topology.[14–16]

Although this model is well supported for N_{cyt} SAs, three sets of recent observations question its applicability to N_{exo} SAs. First, the ER membrane protein complex (EMC) can insert at least some N_{exo} SAs.[10,17] In EMC's absence, these N_{exo} SAs either fail insertion or are inserted in the incorrect orientation. Second, inhibitors of Sec61, which block Sec61's lateral gate,[18] only inhibit insertion of N_{cyt} SAs and have little or no effect on all N_{exo} SAs tested so far.[17,19–21] Third, biochemical or genetic depletion of Sec61 does not impair insertion of N_{exo} SAs.[10,17]

These observations led to speculation that after co-translational targeting of an SA to the ER, those intended for an N_{exo} topology are inserted by EMC whereas N_{cyt} SAs are inserted by Sec61[22,23] (Figure S1B). This view is attractive because EMC is widely conserved across eukaryotes[24] and its core functional subunit (EMC3) is part of the ancient Oxa1 family of insertases present in the last universal common ancestor.[25,26] Oxa1 family members participate in membrane protein topogenesis in bacteria and endosymbiotic organelles.[27]

Although appealing, several aspects of this model remain unclear. First, EMC would need to act co-translationally on

© 2023 MRC Laboratory of Molecular Biology. Published by Elsevier Inc.
This is an open access article under the CC BY license (http://creativecommons.org/licenses/by/4.0/).

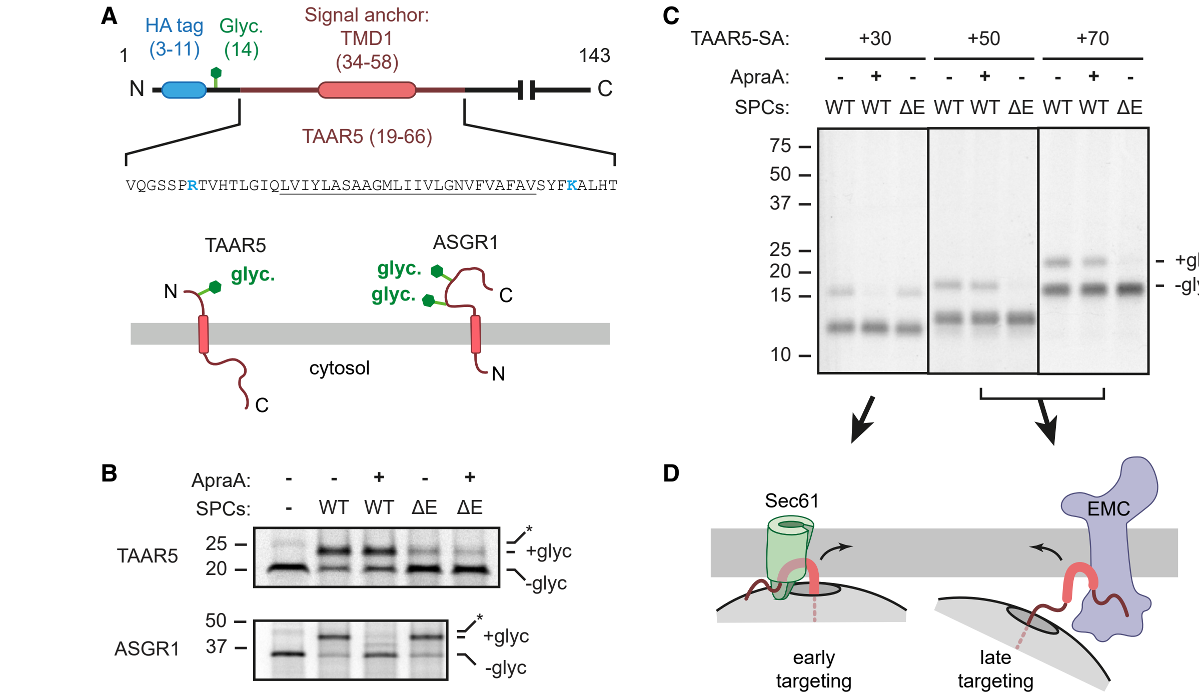

Figure 1. Timing of targeting influences the signal-anchor insertion pathway

(A) Diagrams of the N_{exo} TAAR5 SA reporter cassette (TAAR5-SA). The TMD (underlined) and its flanking sequences are preceded by an N-terminal HA tag and glycosylation site (Glyc.) and followed by a C-terminal segment of unstructured polypeptide. The expected N_{exo} topology of the TAAR5 reporter and expected N_{cyt} topology of human ASGR1 are depicted below.

(B) ^{35}S-methionine labeled TAAR5-SA and ASGR1 were translated in rabbit reticulocyte lysate (RRL) in the absence or presence of SPCs derived from wild type (WT) or ΔEMC (ΔE) HEK293 cells. The Sec61 lateral gate inhibitor Apratoxin A (ApraA) was included where indicated. After translation, the SPCs were recovered by centrifugation and analyzed by SDS-PAGE and autoradiography. An aliquot of the sample lacking SPCs was analyzed directly. The glycosylated (+glyc) and non-glycosylated (−glyc) products are indicated. Asterisk indicates a product seen in samples lacking SPCs and may represent ubiquitin-modified substrate.

(C) ^{35}S-methionine labeled TAAR5 RNCs stalled 30, 50, or 70 aa after the SA were incubated with WT or ΔEMC (ΔE) SPCs in the absence or presence of ApraA. The +glyc and −glyc products are indicated. Note that prior to SDS-PAGE, tRNA was digested from the nascent chain using RNase A.

(D) Model of insertion for early- versus late-targeting RNCs.

See also Figure S1.

SAs, but has not been observed in proximity to the nascent chain, the ribosome, or the Sec61 complex at the key step of SA insertion. Second, EMC's large cytosolic domain means its membrane domain cannot be close to the ribosome exit tunnel.[28] Yet, stalled ribosome-nascent-chain complexes (RNCs) can be targeted to Sec61 even before the entire SA has emerged from the ribosome.[14] This would suggest that SAs fully emerge when the ribosome is already docked at Sec61. Third, because the Sec61-bound ribosome is very close to the membrane,[29] EMC cannot approach within ~100 Å of either Sec61 or the ribosome exit tunnel.[28]

These steric and temporal constraints argue against a direct role for EMC at the early stages of membrane topogenesis. Yet, EMC-dependent N_{exo} SAs can establish their final topology shortly after their emergence from the ribosome.[10] Although one might be tempted to posit that EMC's role in topogenesis is indirect, a direct role in topogenesis is firmly established for the broader Oxa1 family,[27] and EMC can directly facilitate TMD insertion in reconstitution studies.[10,30,31] To address these critical issues of membrane protein biogenesis, we investigated the relationship between Sec61 and EMC during the earliest stages of SA targeting and insertion using *in vitro* reconstitution of these steps combined with genetic and pharmacologic perturbations of the key machinery.

RESULTS AND DISCUSSION

The substrate used for the majority of our analysis is the N_{exo} SA from trace-amine-associated receptor 5 (TAAR5), a G-protein-coupled receptor inserted by EMC.[10] The SA and flanking segments were placed in a reporter cassette containing a glycosylation site and epitope tag near the N terminus (Figure 1A). The SA was followed by a C-terminal cytosolic domain. The overall length (25 aa) and hydrophobicity [ΔG_{app} = −1.5 (Hessa et al.[32])] of the SA would predict an N_{exo} topology,[33] as in native TAAR5. The single positively charged residue on either side of the SA could be consistent with either topology. The N-tail of our reporter (33 aa) is typical of N-tails in N_{exo} SAs,[10] including the 34-aa native TAAR5 N terminus. This substrate, unremarkable from the standpoint of the SA or flanking features, provided a model to probe the relationship between EMC and Sec61 during SA translocation.

CellPress
OPEN ACCESS

The timing of targeting dictates the SA insertion mechanism

In vitro translation of ^{35}S-labeled TAAR5-SA in the presence of semi-permeabilized cells (SPCs) resulted in glycosylation, a reliable proxy for translocation in the correct N_{exo} topology (Figure 1B).[10] The Sec61 inhibitor Apratoxin A (ApraA) had minimal effect on TAAR5-SA translocation but completely inhibited translocation of the model N_{cyt} SA asialoglycoprotein receptor (ASGR1). By contrast, in EMC6-knockout SPCs, called ΔEMC hereafter because the remainder of the EMC is also destabilized,[34] N_{exo} translocation of TAAR5-SA was sharply reduced whereas ASGR1 translocation was unaffected. Notably, the residual N_{exo} insertion seen in ΔEMC SPCs remained refractory to ApraA inhibition. These data indicate that TAAR5-SA is strongly EMC dependent and does not use Sec61's lateral gate for N_{exo} insertion in the absence of EMC. To the extent EMC-independent insertion is seen, this can proceed even when Sec61's lateral gate is blocked, perhaps by unassisted insertion or by using a currently unappreciated factor.

Similar results were observed for TAAR5-SA RNCs stalled 50 or 70 aa downstream of the SA (referred to as TAAR5-SA+50 and TAAR5-SA+70, respectively), albeit at lower overall efficiency of translocation (Figure 1C). Here, translation is stalled at a defined site using rare codons, the RNCs purified by sucrose-gradient fractionation (Figure S1C) and incubated for 10 min with SPCs to study targeting and translocation in a synchronized manner. With ~30 aa buried inside the ribosome, these intermediates have the entire SA exposed outside the ribosome with a tether to the ribosome surface of either ~20 or ~40 aa. Translocation of SA+50 and SA+70 RNCs mimics the co-translational translocation requirements of TAAR5-SA (Figure 1B): strongly EMC dependent and mostly Sec61 independent.

Surprisingly, the opposite result was seen for SA+30 RNCs (Figure 1C). Here, translocation was strongly inhibited by ApraA and impervious to EMC knockout. This intermediate has the complete SA just outside the ribosome exit tunnel, a length at which effective insertion is first seen for N_{exo} SAs.[14] This result indicates that the TAAR5-SA is capable of using Sec61's lateral gate for insertion. Despite this capacity to use Sec61, both the co-translational insertion reaction and the insertion reactions with SA+50 and SA+70 RNCs show very poor insertion into ΔEMC SPCs, and the insertion that is seen is not inhibited by ApraA.

Thus, use of Sec61 for TAAR5 SA insertion in the N_{exo} topology is possible only at a short tether length (Figure 1D, left), at which stage the RNC evidently cannot use EMC. With a longer tether length, the SA does not use Sec61 and instead relies on EMC (Figure 1D, right). These observations imply that under co-translational conditions, TAAR5 RNCs must arrive at Sec61 when at least ~50 aa have been synthesized downstream of the SA, even though targeting at earlier lengths is clearly possible for stalled RNCs.[14,35] If co-translational targeting were to occur early (e.g., when the first half of the SA emerges from the ribosome), the SA could use the Sec61 lateral gate, would not be dependent on EMC, and would be inhibited by ApraA, as seen for SA+30 RNCs (Figure 1C).

EMC-dependent insertion relies on late targeting to Sec61

To test the idea of "late" targeting, we modified the sequence downstream of an N_{cyt} ASGR1 SA such that translocation of its C-terminal tail can occur only if SA targeting to Sec61 occurs early. This was accomplished by placing a 29-aa zinc finger (ZNF) immediately after the SA. If the SA engages Sec61 early, before the last of these 29 aa has emerged into the mouth of the ribosome exit tunnel, ZNF cannot fold because residues needed for Zn^{2+} coordination have already been pulled into the translocation channel (Figure 2A). By contrast, emergence of the entire ZNF before SA engagement of Sec61 would result in rapid Zn^{2+}-dependent folding,[36] which can occur in the mouth of the exit tunnel.[37] The folded domain would block C-terminal translocation,[38] which we assayed by C-tail glycosylation.

C-tail translocation was strongly impaired in a ZNF- and Zn^{2+}-dependent manner only when placed immediately after the SA (Figure 2A). Translocation was unaffected in the absence of Zn^{2+}, which is required for stable ZNF folding, and Zn^{2+} had no effect in the absence of ZNF. Importantly, ZNF had no effect on translocation when placed further downstream. By the time this downstream ZNF is synthesized, C-tail translocation is already in progress. Hence, the initial segment of ZNF enters Sec61 as it emerges from the ribosome, never getting a chance to fold in the cytosol. Conversely, ZNF immediately downstream of the SA must have fully emerged into the mouth of the exit tunnel before the SA engages Sec61. Similar results were seen when ZNF was placed downstream of a cleavable signal sequence *in vitro* and in cells.[38]

From the length of ZNF (29 aa) and dimensions of the ribosome exit tunnel (~25 aa from the peptidyl-transferase center to the wide part at the mouth), we infer that at least ~54 aa are synthesized downstream of the SA without having initiated translocation. Other systems to monitor the timing of targeting in mammalian cells and in yeast similarly indicate that between 40 and 100 aa downstream of the SA had emerged from the ribosome before the initiation of translocation at Sec61.[40,41] Yet, experiments with stalled RNCs show that N_{cyt} engagement of Sec61 can occur with a downstream tether of only 39 aa and reaches maximal efficiency with a 44 aa tether.[42] Similar results were seen with an N_{exo} SA using a series of stalled RNCs.[14,35]

We conclude that although an SA or signal peptide is capable of engaging Sec61 very early under elongation-arrested conditions, engagement under conditions of continuous translation seems to occur notably later. Partial ZNF-mediated inhibition was still seen when ZNF was placed up to 15 aa downstream of the SA, indicating that Sec61 engagement is not complete even when ~69 downstream aa have been synthesized (Figure S2A). Because the same results were seen in ΔEMC SPCs (Figure S2A), we infer that the reason for late targeting is not due to EMC somehow slowing RNC transfer from SRP to Sec61 but is rather an intrinsic feature of the targeting system.

Late SA delivery to Sec61 would explain why effective N_{exo} insertion via Sec61's lateral gate, which requires a very short RNC (Figure 1C), cannot occur in the absence of EMC (Figure 1B). Furthermore, it allows for the possibility that EMC-dependent SA translocation, which can occur for SA+50 RNCs (Figure 1C), might happen before RNC delivery to Sec61, at

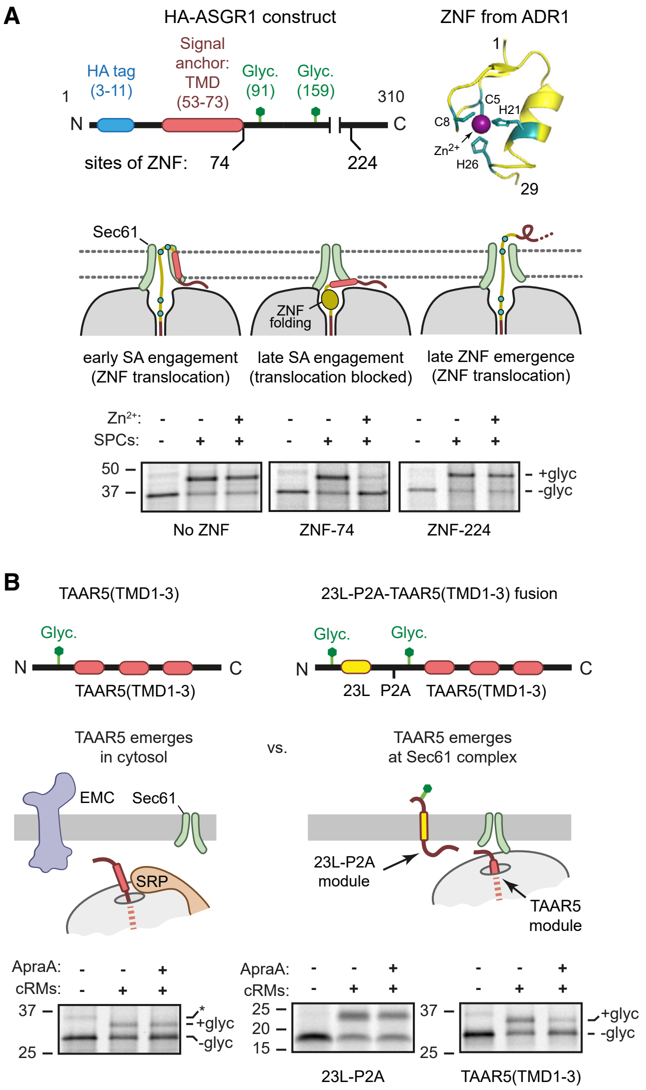

Figure 2. EMC-dependent signal-anchor insertion relies on late targeting

(A) Top: diagram of HA-ASGR1 containing or lacking a zinc finger from ADR1 (ZNF) immediately after the SA (ZNF-74) or after 150aa (ZNF-224). The folded ZNF structure (PDB: 2ADR[39]) is shown with key residues required for Zn^{2+}-dependent folding in teal. Diagram showing how timing of targeting influences ZNF folding and HA-ASGR1 translocation. Bottom: the HA-ASGR1 constructs from the diagram were translated in the absence or presence of SPCs and Zn^{2+} where indicated. After translation, the SPCs were recovered by centrifugation and analyzed by SDS-PAGE and autoradiography. An aliquot of the sample lacking SPCs was analyzed directly. The glycosylated (+glyc) and non-glycosylated (−glyc) products are indicated.

(B) Top: construct design and a diagram depicting the situation where the substrate emerges from a cytosolic versus Sec61-associated ribosome. Bottom: ^{35}S-methionine labeled TAAR5(TMD1-3) or 23L-P2A-TAAR5(TMD1-3) were translated in the absence or presence of canine-pancreas-derived rough microsomes (cRMs). Sec61 inhibitor ApraA was included where indicated. The TAAR5(TMD1-3) protein was recovered by IP via the N-terminal HA tag. The 23L-P2A fragment was visualized by direct analysis of total translation reactions. The +glyc and −glyc products are indicated. Asterisk indicates a product seen in samples lacking SPCs and may represent ubiquitin-modified substrate.

See also Figure S2.

which point EMC would be sterically prevented from approaching the exit tunnel.[28] To test whether late delivery to Sec61 is a requirement for EMC-dependent SA translocation, we engineered a situation where the SA emerges from a Sec61-docked ribosome, thereby ensuring early delivery (Figure 2B). To do this, we placed an artificial, highly efficient N_{exo} SA (23 leucine residues), a ~100 aa spacer, and a viral-derived P2A sequence in front of a TAAR5 reporter (the first three TMDs of TAAR5). We reasoned that the TAAR5 reporter would be synthesized after the 23L-P2A module had already mediated targeting and docking at Sec61. Importantly, the P2A sequence causes peptide bond skipping,[43] so TAAR5 is synthesized as a separate protein by a Sec61-bound ribosome.

When pre-targeted to Sec61 in this way, we found that correct TAAR5 insertion became sensitive to ApraA, being inhibited by at least ~50% (Figure 2B). Note that the 23L-P2A module was not inhibited by ApraA, so the effect on TAAR5 is not due to effects at an earlier step. The identical TAAR5 reporter that was not preceded by the 23L module was resistant to ApraA (Figure 2B) and strongly dependent on EMC (Figure S2B). This illustrates that enforcing early TAAR5-SA targeting to Sec61 permits N_{exo} insertion via the Sec61 lateral gate. Notably, alternative insertion routes (e.g., via EMC) apparently cannot be accessed effectively by early-targeted RNCs under conditions when Sec61 is inhibited. Thus, EMC-dependent SA insertion relies on late targeting to Sec61, occurring after more than 50 downstream aa have been produced. At this length, the RNC is competent for EMC-dependent N_{exo} insertion and inefficient at using Sec61 (Figure 1C).

Crosslinking reveals EMC interaction with substrate

A key prediction of the findings so far is that RNCs should transiently be adjacent to EMC at early stages of biogenesis. To investigate this, we analyzed insertion intermediates by chemical crosslinking. We produced sucrose-gradient-purified ^{35}S-labeled TAAR5-SA+70 RNCs containing a single cysteine in the N-tail and used cysteine-reactive crosslinking with bismaleimidohexane (BMH). Incubation of these RNCs with SPCs produced a mixture of membrane-bound targeting intermediates that crosslink to SRP54 and fully translocated products that crosslink to an ER lumenal protein (Figure S3A). In ΔEMC SPCs, the SRP54 crosslink was unchanged, consistent with SRP acting before EMC (Figure S3A). By contrast, crosslinks at later steps differed: the glycosylated product and lumenal crosslink were sharply reduced and a crosslink to the single cytosolic cysteine in Sec61β was enhanced. Thus, the substrate's N-tail faces the cytosol, consistent with these Sec61-docked RNCs having failed N_{exo} translocation. Although this result suggests that EMC acts between SRP and Sec61, no clear crosslinks to EMC subunits were evident.

To enrich for potential weak EMC crosslinks, we replaced endogenous EMC3 (to ~70%–90%) with a FLAG-tagged version by long-term stable overexpression. Under these conditions, all excess EMC3 is degraded by quality control (Figure 3A, top left). Immunoprecipitation (IP) of BMH crosslinking products from EMC3-FLAG SPCs via the FLAG tag under non-denaturing conditions revealed a weak but specific product that was not seen in ΔEMC SPCs (Figure S3A). Based on its molecular weight, the

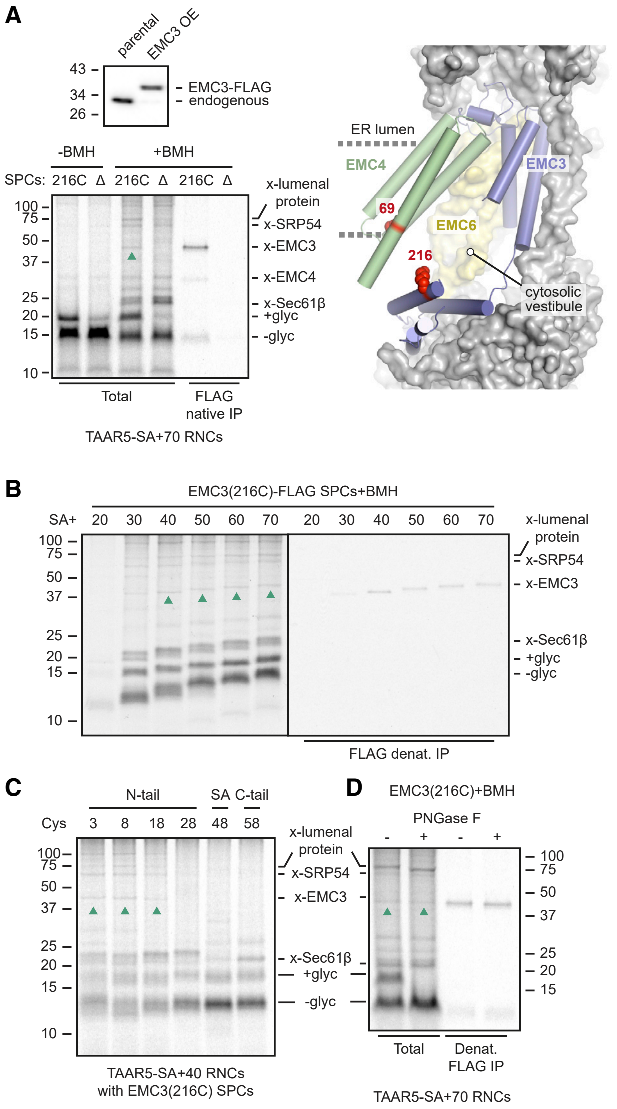

Figure 3. Detection of substrate-EMC interactions by crosslinking

(A) Top: anti-EMC3 immunoblot of cells stably overexpressing EMC3-FLAG compared with parental cells. Bottom: ^{35}S-methionine labeled TAAR5-SA+70-RNCs were incubated with SPCs containing EMC3(216C)-FLAG or lacking EMC (Δ). One aliquot was analyzed directly (−BMH) and another treated with BMH (+BMH). The crosslinked products were analyzed directly or after native IP of EMC3 via the FLAG tag. The non-glycosylated (−glyc) and glycosylated (+glyc) products and crosslinks to Sec61β, EMC4, EMC3 (upward green arrows), SRP54, and an ER lumenal protein are indicated. Prior to SDS-PAGE, tRNA was digested from the nascent chain using RNase A. Right: structural model of EMC (PDB: 6WW7,[44] 6Z3W[28]) showing positions of crosslinked residues in EMC3 (216) and EMC4 (69).

(B) ^{35}S-methionine labeled TAAR5 RNCs of indicated lengths were incubated with EMC3(216C)-FLAG SPCs, subjected to BMH crosslinking, and analyzed directly or after denaturing EMC3 IP via the FLAG tag.

(C) ^{35}S-methionine labeled TAAR5-SA+40 with single cysteines at the indicated positions were incubated with EMC3(216C)-FLAG SPCs, crosslinked with BMH, and analyzed directly.

(D) The BMH crosslinking reaction of ^{35}S-methionine labeled TAAR5-SA+70 RNCs (as in A) was digested with PNGase F where indicated and analyzed directly (total) or after denaturing IP of EMC3-FLAG.

See also Figure S3.

presence of a semi-buried cysteine in EMC4 facing the cytosol (Figure 3A, right), and the absence of any other accessible cysteines in the cytosolic or intramembrane regions that might participate in N-tail translocation, this weak crosslink was assigned to EMC4.

We exploited the absence of other accessible EMC cysteines to engineer a single cysteine at position 216 of EMC3, located at the entrance to the cytosolic vestibule through which substrates might pass in one model of EMC function.[28,44–46] Using SPCs from cells stably expressing this 216C variant of EMC3, BMH crosslinking to TAAR5-SA+70 RNCs with a cysteine in the N-tail showed a single new product relative to wild-type or ΔEMC3 SPCs (Figure 3A, bottom-left; compare with Figure S3A). The new product was a crosslink to EMC3-FLAG as judged by its size, dependence on 216C, and IP by anti-FLAG antibodies. The fainter smaller product in the native anti-FLAG IP is likely the same EMC4 crosslink seen in Figure S3A.

Using BMH crosslinking to EMC3(216C), we found that EMC3 is prominently adjacent to TAAR5-SA+40 and longer intermediates, the translocation of which are all EMC dependent (Figure S3B). The slightly shorter SA+30 intermediate does not crosslink as effectively to EMC3 (although a non-EMC product of very similar size is seen (Figure S3C)], consistent with its translocation by an EMC-independent and Sec61-dependent mechanism (Figures S3B and 1C). The even shorter SA+20 intermediate, in which the SA is only partially emerged from the ribosome, was poorly targeted to the SPCs and showed an overall weak signal.

Crosslinking to EMC3(216C) was seen from each of three sites along the N-tail (at positions 3, 8, and 18) but was sharply diminished from sites adjacent to, within, or beyond the SA (Figure 3C). The EMC sampling step is transient, after which all RNCs dock on Sec61, explaining why crosslinks to Sec61β are more prominent than to EMC3 crosslinks. All crosslinks formed rapidly (Figure S3D), indicative of direct physical proximity rather than progressive trapping of non-specific collisional interactions among abundant proteins. Consistent with the cytosolic positioning of residue 216 in EMC3, the crosslinked substrate was not glycosylated and did not shift upon N-glycanase digestion (Figure 3D). This is in contrast to the glycosylated substrate and its crosslink to a lumenal protein. The findings suggest that the EMC-substrate interaction represents a pre-translocation state when the N-tail is at the cytosolic vestibule in front of EMC3.

Substrate location within EMC before translocation

Single cysteines at ten additional positions in EMC3 were tested for proximity to the N-tail of TAAR5-SA+40, the shortest RNC for which EMC-dependence is seen. Residues 63, 216, and 223 yielded the strongest crosslinks, residues 101 and 240 yielded somewhat weaker crosslinks, and minimal crosslinks were seen from six other positions (Figure S4A). Mapping the EMC3 crosslink sites onto a structural model of EMC revealed that the three strongest crosslinking positions are close to each other, with the two weaker crosslinking positions flanking either side (Figure 4A). The center of this substrate-interacting hub is at the entry to EMC's cytosolic vestibule leading to an intramembrane hydrophilic groove in EMC3 defined as its front side.

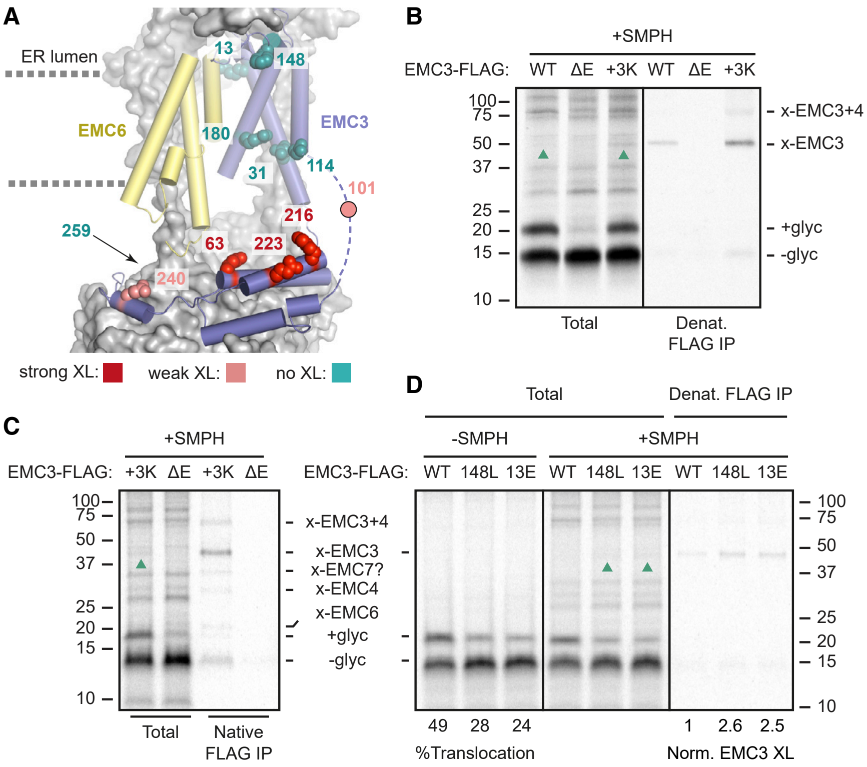

Figure 4. Mapping substrate location at EMC

(A) EMC3 positions that crosslink strongly (red), weakly (pink), or minimally (teal) to the N-tail of TAAR5 RNCs (see Figure S4) are mapped onto the EMC structure (PDB: 7ADO[46]).

(B) SMPH Crosslinking reactions between ^{35}S-methionine-labeled TAAR5-SA+70 and SPCs stably expressing EMC3-FLAG (WT) or EMC3(63K/213K/223K)-FLAG (3K) versus SPCs lacking EMC (ΔE). Samples were analyzed directly (total) or after denaturing IP of EMC3-FLAG. The non-glycosylated (−glyc) and glycosylated (+glyc) products and crosslinks to EMC3 (upward green arrows) are indicated. A three-way crosslink between substrate, EMC3, and EMC4 was also observed at low levels.

(C) Experiment as in (B) but immunoprecipitated under native conditions, revealing EMC crosslinks consistent with EMC4, EMC6, and EMC7.

(D) Experiment as in (B) but using SPCs stably expressing wild-type EMC3-FLAG, EMC3(148L)-FLAG, or EMC3(13E)-FLAG.

See also Figure S4.

This groove is shared among all Oxa1 family insertases and is thought to facilitate translocation of a hydrophilic flanking domain concomitant with TMD insertion.[3] The absence of crosslinks from three sites that line EMC3's groove suggest that the actual translocation reaction is rapid relative to the pre-translocation sampling step.

To obtain further information about the EMC-substrate interaction, we also analyzed crosslinking to lysine side chains in EMC using the amine-sulfhydryl hetero-bifunctional crosslinker succinimidyl 6-((beta-maleimidopropionamido)hexanoate) (SMPH). Although lysines are widely distributed across EMC, the efficiency of crosslinking was low and only detected after IP of EMC3 (Figure S4B). This was the case even though position 216, from which cysteine-reactive crosslinking is readily evident, ordinarily contains a lysine. It seems that the amine-reactive NHS ester is less efficient than sulfhydryl-reactive maleimide under our experimental conditions. When three additional lysine residues are introduced in this region (at positions 63, 213, and 223), crosslinking efficiency increases to the point EMC3 crosslinks are evident without IP (Figure 4C).

Non-denaturing purification of the crosslinking products via EMC3 did not recover any products of greater prominence than the EMC3 crosslink from either wild type EMC3 (Figure S4B) or from the lysine-supplemented EMC3-containing complex (Figure 4C). Because these purification conditions recover all nine EMC subunits (see Figure S4C), these results further suggest that the subunit of nearest proximity to these RNCs is probably EMC3. Crosslinking to EMC6, EMC4, and possibly EMC7 (assigned on the basis of size) were also seen, whereas crosslinks to EMC5 and EMC1 were not. EMC4 and EMC7 are on the front side of the EMC3-EMC6 module, whereas EMC5 and EMC1 are on the back, providing support to the idea that substrate engages EMC via the front-side vestibule. This matches the BMH crosslinking to EMC4 via its cytosolic semi-buried cysteine near the vestibule (Figure S3A).

The EMC-substrate interaction captured by crosslinking is likely a pre-translocation intermediate. If so, one might expect this intermediate to be populated to a greater extent by EMC3 mutants that partially impair substrate translocation.[44,46] Using SMPH crosslinking of TAAR5-SA+70 RNCs with a cysteine in the center of the N-tail, we found that two different EMC3 mutants showed increased crosslinks to substrate concomitant with reduced translocation (Figure 4D). Importantly, the mutants, which are at the EMC3-EMC6 interface and the EMC3-EMC4 interface, do not impair the assembly or abundance of EMC (Figure S4C).

The crosslinking experiments provide the first direct evidence for a substrate-EMC interaction during the SA insertion reaction under native conditions. The earlier interactions with substrate were with isolated TMDs and purified EMC in detergent,[44] or with sub-domains of EMC.[28,44] Based on EMC crosslinking to pre-translocation RNCs, the site within EMC that is being sampled, steric considerations, and the lengths at which crosslinking is seen, we infer that the step we are observing probably occurs between SRP-mediated targeting and RNC docking at Sec61.

EMC acts after SRP release and before Sec61 docking

To test our placement of EMC between SRP and Sec61, we monitored substrate-EMC interactions under conditions where the SRP or Sec61 steps are perturbed. Omitting GTP or including the slowly hydrolyzed GTP analog guanylyl-(alpha, beta)-methylene-diphosphonate (GMPCPP) during the insertion reaction completely or partially precluded SA translocation as judged

CellPress
OPEN ACCESS

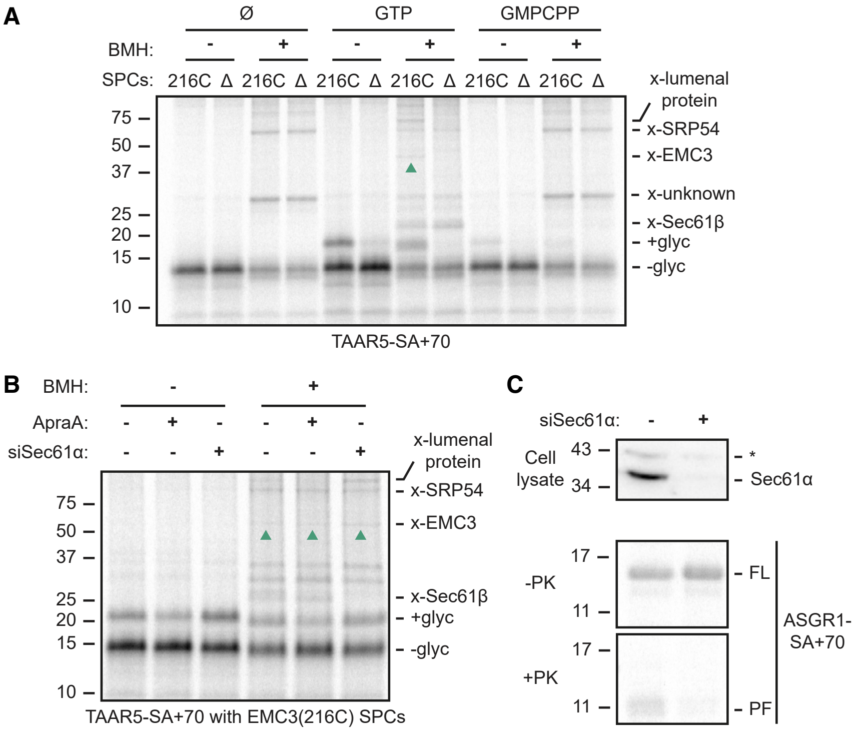

Figure 5. EMC acts between SRP and Sec61

(A) ^{35}S-methionine labeled TAAR5-SA+70 RNCs were mixed with GTP, the slowly hydrolyzed GTP analog GMPCPP or nothing (Ø), then incubated with SPCs containing EMC3(216C)-FLAG or lacking EMC (Δ). Pelleted SPCs were subject to BMH crosslinking as indicated and analyzed directly. The non-glycosylated (−glyc) and glycosylated (+glyc) products and crosslinks to Sec61β, EMC3 (upward green arrows), SRP54, and a lumenal protein are indicated.

(B) ^{35}S-methionine-labeled TAAR5-SA+70 were incubated with SPCs stably expressing EMC3(216C)-FLAG that had been treated with control siRNA or siRNA targeting Sec61α. Sec61 inhibitor ApraA was included where indicated. After crosslinking with BMH as indicated, the samples were analyzed by SDS-PAGE and autoradiography.

(C) Top: immunoblot showing the extent of Sec61α knockdown. Bottom: the control or Sec61-knock-down SPCs were incubated with ASGR1-SA+70 RNCs and either analyzed directly (−PK) or after digestion with proteinase K (+PK). The full-length (FL) ASGR1 product and protease-protected fragment (PF) of ASGR1 are indicated.

See also Figure S5.

by N-tail glycosylation (Figure 5A). This is expected because dissociation of SRP from its receptor and substrate are dependent on GTP hydrolysis.[47] Hence, crosslinks to SRP54 are enhanced under these conditions, whereas crosslinks to EMC, Sec61β, and lumenal protein are sharply decreased (Figure 5A). Placing the substrate cysteine in two other positions within its N-tail generated similar results (Figure S5). Thus, substrate release from SRP is essential for the N-tail to be within crosslinking distance of EMC.

Crosslinking analysis in SPCs from transient Sec61 knockdowns or Sec61 inhibition with ApraA showed little or no effect on either N-tail translocation or EMC interaction by crosslinking (Figure 5B). The knockdown efficiency was more than ~80% and sufficient to have a strong effect on insertion of the N_{cyt} SA from ASGR1 as assayed by protease protection (Figure 5C). These results indicate that immediately after SRP-mediated targeting, the substrate's N-tail, although near the membrane and able to sample a fairly large radius (e.g., when the Cys is close to the N terminus), cannot interact with EMC. Only upon GTP-dependent SA release from SRP is the N-tail found close to the cytosolic vestibule of EMC. This step is not dependent on Sec61, excluding a model where EMC must act on Sec61-docked RNCs. Rather, the reduced translocation of an EMC-dependent SA when it emerges at an inhibited Sec61 (Figure 2B) argues that ribosome docking at Sec61 precludes access to EMC, consistent with previously noted steric limitations.[28]

N-tail charge influences substrate residence at EMC

The human genome contains ~2,600 SAs with N-tails short enough (<100 aa) to potentially allow insertion in either N_{exo} or N_{cyt} orientation. Using site-specific crosslinking between EMC3 (216C) and an N-tail cysteine, we observed that RNCs of the N_{cyt} protein ASGR1 crosslink to EMC3 when stalled 70 or 85 aa downstream of the SA (Figure 6A). The longer RNC shows that N_{cyt} insertion was not affected by EMC deletion as judged by C-tail glycosylation. Two other N_{cyt} SAs of unrelated proteins similarly crosslinked to EMC as RNCs (Figure S6A). This suggests a model in which all SAs sample EMC, with only a subset of them inserting via EMC in the N_{exo} topology before Sec61 docking. SAs not inserted by EMC would be inserted by Sec61 in the N_{cyt} topology given its limited capacity for N_{exo} insertion when the SA has a long downstream tether to the ribosome surface (Figures 1C and S3B). Signal sequences probably also sample EMC, with some of them being mis-inserted in the N_{exo} topology before subsequent extraction by ATP13A1.[48]

A key feature of topology determination is charged residues flanking the SA. To place this parameter in the context of our working model, we manipulated flanking charges of the TAAR5 SA (Figure 6B) and examined the consequences for translocation, dependence on EMC and Sec61, and physical interactions with EMC. Removing or reversing the single positive charge in the N-tail led to a modest but clear increase in translocation (Figure 6C). By contrast, the same changes to the single positive charge downstream of the SA had no effect. Focusing on the N-tail charge mutants, we found that although each construct retained strong EMC-dependence (Figure S6B), removing or reversing the positive charge led to slightly increased EMC-independent translocation and slightly decreased crosslinking to EMC3 at its cytosolic vestibule (Figure 6D). Thus, positive charge(s) in the N-tail impedes insertion while increasing residence time in the pre-translocated state.

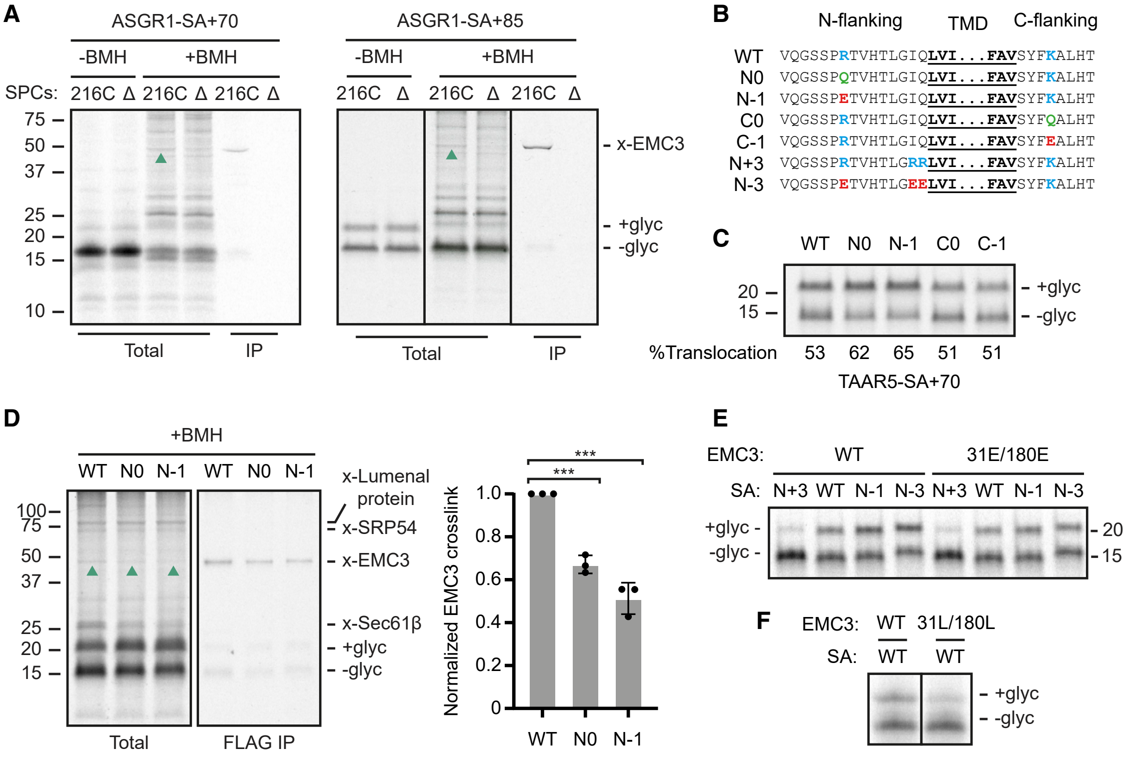

Figure 6. Determinants of EMC-mediated signal-anchor insertion

(A) ^{35}S-methionine labeled RNCs of ASGR1-SA+70 and ASGR1-SA+85 were incubated with SPCs stably expressing EMC3(216C)-FLAG or lacking EMC (ΔE), then subject to BMH crosslinking as Figure 3A. The non-glycosylated (−glyc) and glycosylated (+glyc) products and crosslink to EMC3 (green arrows) are indicated.

(B) Sequence of the mutations analyzed in (C)–(E).

(C) ^{35}S-Methionine-labeled RNCs of the indicated TAAR5-SA+70 variants were incubated with wild-type SPCs and analyzed directly by SDS-PAGE and autoradiography.

(D) BMH crosslinking analysis as in Figure 3, with quantification of relative EMC3 crosslinks (right). Mean ± standard deviation of three independent measurements is plotted. * $p < 0.001$ by one-way ANOVA from Tukey's test.

(E) ^{35}S-methionine-labeled RNCs of the indicated TAAR5-SA+70 variants were incubated with SPCs stably expressing either wild type or EMC3(R31E/R180E)-FLAG and analyzed as in (C).

(F) ^{35}S-methionine-labeled RNCs of TAAR5-SA+70 were incubated with SPCs stably expressing either wild type or EMC3(R31L/R180L)-FLAG and analyzed as in (C). The two samples are from non-adjacent lanes of the same gel exposed for the same period of time from the same experiment.

See also Figure S6.

Because N_{cyt} SAs typically have flanking positive charges in the N-tail, they would be more likely to be "rejected" for N_{exo} insertion by EMC during this sampling step before being transferred to Sec61. Indeed, introducing two extra positive charges just preceding the SA sharply reduced N_{exo} insertion, whereas an acidic N-tail was inserted more efficiently (Figure 6E). Surprisingly, changing two highly conserved arginine residues in EMC3's hydrophilic groove (at positions 31 and 180) to glutamates had no detectable effect on N-tail translocation regardless of the charged residues flanking the substrate's SA (Figure 6E). By contrast, reducing the hydrophilicity of the groove by mutating positions 31 and 180 to leucines impaired translocation (Figure 6F). This argues that the groove does not impose the "positive inside" rule, the nature of which remains to be explored.

Model for signal-anchor topogenesis

We have identified a previously unknown step between SRP-mediated targeting and ribosome docking at Sec61. This new step, which involves direct nascent chain sampling by EMC, plays a key role in determining the topology of proteins that contain a SA close to the N terminus. Although many questions remain, establishing a role for EMC at this point in membrane biogenesis substantially changes the long-standing model for how SA topology is determined. Rather than topology being determined only after RNC delivery to Sec61,[33] we find that EMC transiently samples SAs at an earlier step and inserts a subset of them in the N_{exo} topology. Those SAs that are skipped by EMC arrive at Sec61, which preferentially favors SA insertion in the N_{cyt} topology. Thus, a two-step sequential triage by EMC and Sec61 mediates the insertion and topogenesis of SAs (Figure 7A).

After SRP-mediated targeting to the SRP receptor (SR), the two interacting GTPase domains of SRP54 and SRα can move to a distal site on SRP RNA, creating the "prehandover" complex.[52,53] Although this creates more space around the ribosome exit tunnel, the membrane is still more than 50 Å away and too far for Sec61 to reach its binding site.[49] EMC, which is more

CellPress
OPEN ACCESS

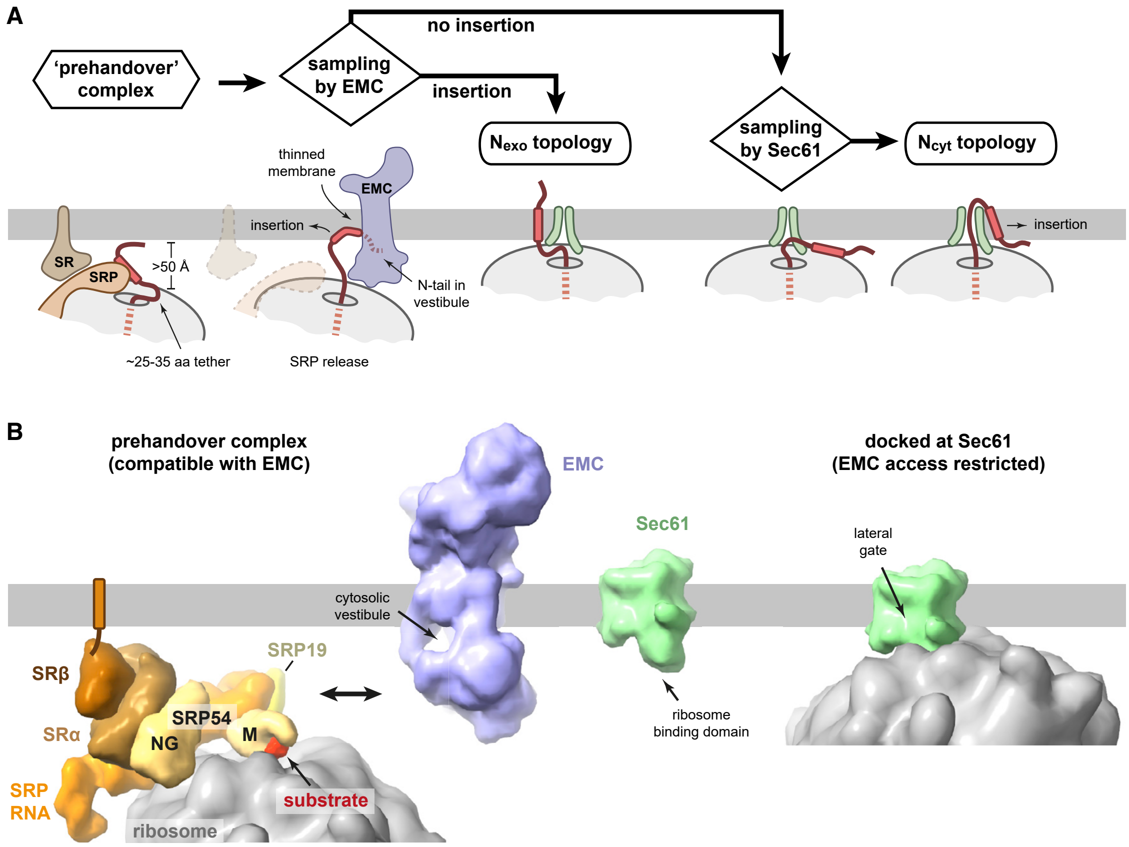

Figure 7. Model for signal-anchor topogenesis

(A) After targeting, the SRP-SR complex rearranges into the prehandover configuration. The large size of the SRP-SR complex precludes the ribosome exit tunnel from approaching the membrane (see B), thereby allowing access to EMC without competition from Sec61. Release of the SA from SRP allows SA binding to the membrane and sampling of EMC's cytosolic vestibule by the N-tail. The SA can reach the membrane because a downstream tether of more than 25 aa has already been synthesized by this point. The EMC sampling step is transient and once the SRP-SR complex dissociates, the ribosome docks at Sec61, at which point access to EMC is restricted due to steric hinderance by the ribosome. If the SA was inserted during the EMC sampling step, the substrate achieves the N_{exo} topology. Otherwise, Sec61 can insert the SA in the N_{cyt} topology.

(B) Scale models illustrating that the large cytosolic domain of EMC can fit between the membrane and ribosome in the prehandover complex (left). By contrast, Sec61's ribosome-binding domain cannot reach its site on the ribosome. After ribosome docking on Sec61 (right), EMC can no longer approach close to the exit tunnel. The prehandover complex is from PDB: 6FRK.[49] EMC is from a composite of PDB: 7ADO,[46] PDB: 6Z3W,[28] and AlphaFold2.[50] The ribosome-Sec61 complex is from PDB: 3J7R.[51]

abundant than SR,[54] can approach the prehandover complex without clashing with the ribosome or competition from Sec61 (Figure 7B, left). GTP hydrolysis by SRP54 and SRα is needed to release the SA from SRP54 and eventual dissociation of SRP from SR, presumably in that order.[47] After release from SRP54, the SA might rapidly bind the membrane at the interfacial region and lie parallel to the lipid bilayer, a reaction that is both fast and highly favored for a hydrophobic domain.[55] The RNC would now be tethered to the membrane, allowing SRP and SR to dissociate without losing the ribosome back to the cytosol. The N-tail of this membrane-tethered RNC can sample the cytosolic vestibule of EMC, which we propose is the state captured by site-specific crosslinking. The time window for EMC sampling might be extended in some cases by clusters of rare codons that have been observed downstream of SAs.[56]

For such an EMC-RNC arrangement, the ribosome surface would need to be separated sufficiently from the membrane surface to provide space for EMC's cytosolic domain. Our ZNF experiments indicate that 54 to 69 aa have already been synthesized before delivery to Sec61, so the pre-Sec61 intermediate might have a tether of 24 to 39 aa between the SA and ribosome surface. It seems that early targeting to Sec61 (just as the SA is emerging from the ribosome) can occur only with a stalled RNC,[14,35] calling into question some of the interpretations derived from such intermediates. For example, SA+30 TAAR5 RNCs can clearly insert in the N_{exo} topology via Sec61's lateral gate, yet this evidently does not occur during a co-translational translocation reaction with full-length substrate.

If the N-tail is short, unstructured, and not enriched in basic residues near the SA, it can be translocated across the membrane by EMC concomitant with the SA transitioning from the interfacial region to the membrane interior. The hydrophilic groove formed by the EMC3-EMC6 module, together with a thinner membrane on this side of EMC,[44] would lower the

energetic barrier to N-tail translocation. This idea is consistent with the finding that a more hydrophobic groove impedes SA insertion (Figure 6F;[44–46]). EMC's role might be dispensable for SA sequences whose partitioning into the membrane is favored relative to the penalty of its N-tail translocation,[55] explaining why some N_{exo} substrates are EMC independent.[10]

Although EMC can mediate post-translational N_{cyt} insertion for tail-anchored proteins with short C-tails, co-translational N_{cyt} insertion by EMC of SAs does not seem to occur. This is probably due to the absence of a membrane-spanning hydrophilic channel in EMC that would be needed to support co-translational translocation of a lengthy hydrophilic polypeptide. EMC also cannot translocate N-tails that are structured or long, presumably for a similar reason. Thus, EMC's architecture, similar to that of all Oxa1 family members, necessarily favors TMDs whose translocated flanking domain, whether the N- or C-tail, are short and unstructured. Why positive charges adjacent to the SA impede translocation is less clear, but unexpectedly, does not rely strongly on repulsion by positive charges in EMC3's groove. Perhaps repulsion additionally requires local positive charges on lipid head groups[57] or involves positive charges on other regions of EMC. The same repulsion mechanism probably explains why mitochondrial TA proteins, which often contain positively charged tails, are disfavored from ER insertion by EMC.[48,58]

The EMC sampling step is probably brief, after which the RNCs are pulled very close to the membrane by binding to Sec61 (Figure 7B, right). EMC would no longer have access to the nascent chain (unless the nascent chain has an extremely long tether), explaining why an SA that emerges from a Sec61-bound ribosome cannot use EMC for insertion. Although Sec61 is capable of mediating N_{exo} insertion via its lateral gate, this can only occur at a very early stage of elongation when the SA has just emerged from the ribosome. Because RNCs arrive at Sec61 at a later stage than this, Sec61-mediated insertion favors the N_{cyt} topology.

Our findings lead to a two-stage mechanism for SA insertion: EMC operates first and can mediate N_{exo} insertion, whereas Sec61 operates second to mediate N_{cyt} insertion of those SAs skipped by EMC. Although prokaryotes do not have EMC, they have YidC, another Oxa1 family member.[27] The same two-stage mechanism might also operate in prokaryotes, consistent with the proximity of YidC to SRP and its receptor.[59,60] EMC's role in N_{exo} SA insertion explains why N_{exo} membrane proteins are refractory to Sec61 inhibitors.[17,19–21] Because EMC acts first, some N_{cyt} SAs and signal peptides might be inappropriately inserted in the N_{exo} topology by EMC before Sec61 even has a chance. This explains why cells have evolved a widely conserved dislocation system to specifically correct such defects.[61]

Despite EMC being abundant and acting between two very-well-studied steps in protein targeting, its existence was overlooked in earlier crosslinking studies. One reason might be that most earlier work focused on interactions made by the SA (or signal peptide), whereas EMC is proximal to the substrate N-tail to be translocated. Furthermore, the transient nature of EMC's function, the apparent absence of stable ribosome binding, and poor crosslinking efficiency unless reactive residues are positioned at certain positions might have contributed to its invisibility. With clear evidence for its direct role in SA insertion, placement within the broader framework of protein targeting, and reconstitution of these events *in vitro*, the way is now open to mechanistic and structural analysis.

Limitations of the study

Beyond the many open questions for future work noted in the discussion, the study has two important limitations. First, the conclusions are based on the analysis of protein translocation reconstituted in a cell-free system. Although this system has a long history of faithfully recapitulating many aspects of translocation *in vivo*, one important difference is the slower kinetics of biochemical reactions, including a severalfold slower translation rate. A dynamic co-translational process may therefore operate somewhat differently. Second, much of the study relies on stalled RNCs which probably contain a mixture of on-pathway intermediates and off-pathway products. Our assignments of on-pathway intermediates could be incorrect in some cases, resulting in different interpretations to those we have proposed.

STAR★METHODS

Detailed methods are provided in the online version of this paper and include the following:

- KEY RESOURCES TABLE
- RESOURCE AVAILABILITY
  - Lead contact
  - Materials availability
  - Data and code availability
- EXPERIMENTAL MODEL AND SUBJECT DETAILS
  - Cell lines
- METHOD DETAILS
  - DNA
  - Generation of stable cell lines
  - Knockdown with siRNA
  - Preparation of semi-permeabilized cells
  - *In vitro* transcription and translation
  - Isolation of ribosome nascent chain complexes (RNCs)
  - *In vitro* insertion assays with purified RNCs
  - Protease protection assays with purified RNCs
  - Site-specific chemical crosslinking
  - Immunoprecipitation
  - Affinity purification of EMC
  - SDS-PAGE and western blotting
- QUANTIFICATION AND STATISTICAL ANALYSIS
  - Quantification of autoradiographs
  - Statistics
  - Reproducibility

SUPPLEMENTAL INFORMATION

Supplemental information can be found online at https://doi.org/10.1016/j.molcel.2023.01.018.

ACKNOWLEDGMENTS

We thank L. Smalinskaitė, A. Lewis, H. Wang, and S. Juszkiewicz for productive discussions; and L. Smalinskaitė and A. Lewis for comments on this manuscript. This work was supported by the UK Medical Research Council (grant

CellPress
OPEN ACCESS

MC_UP_A022_1007 to R.S.H.). H.W. was supported by EMBO Postdoctoral Fellowship.

AUTHOR CONTRIBUTIONS

H.W. performed all experiments in the study. H.W. and R.S.H. conceived the project, and R.S.H. provided guidance and mentoring. H.W. and R.S.H. wrote the paper.

DECLARATION OF INTERESTS

R.S.H. is a member of the advisory board for *Molecular Cell*.

Received: October 3, 2022
Revised: December 8, 2022
Accepted: January 18, 2023
Published: February 9, 2023

REFERENCES

1. Krogh, A., Larsson, B., von Heijne, G., and Sonnhammer, E.L. (2001). Predicting transmembrane protein topology with a hidden Markov model: application to complete genomes. J. Mol. Biol. *305*, 567–580. https://doi.org/10.1006/jmbi.2000.4315.
2. von Heijne, G. (2007). The membrane protein universe: what's out there and why bother? J. Intern. Med. *261*, 543–557. https://doi.org/10.1111/j.1365-2796.2007.01792.x.
3. Hegde, R.S., and Keenan, R.J. (2022). The mechanisms of integral membrane protein biogenesis. Nat. Rev. Mol. Cell Biol. *23*, 107–124. https://doi.org/10.1038/s41580-021-00413-2.
4. Blobel, G. (1980). Intracellular protein topogenesis. Proc. Natl. Acad. Sci. USA *77*, 1496–1500. https://doi.org/10.1073/pnas.77.3.1496.
5. Spiess, M., Junne, T., and Janoschke, M. (2019). Membrane protein integration and topogenesis at the ER. Protein J. *38*, 306–316. https://doi.org/10.1007/s10930-019-09827-6.
6. Beltzer, J.P., Fiedler, K., Fuhrer, C., Geffen, I., Handschin, C., Wessels, H.P., and Spiess, M. (1991). Charged residues are major determinants of the transmembrane orientation of a signal-anchor sequence. J. Biol. Chem. *266*, 973–978.
7. Nilsson, J., Persson, B., and von Heijne, G. (2005). Comparative analysis of amino acid distributions in integral membrane proteins from 107 genomes. Proteins *60*, 606–616. https://doi.org/10.1002/prot.20583.
8. von Heijne, G., and Gavel, Y. (1988). Topogenic signals in integral membrane proteins. Eur. J. Biochem. *174*, 671–678. https://doi.org/10.1111/j.1432-1033.1988.tb14150.x.
9. Wahlberg, J.M., and Spiess, M. (1997). Multiple determinants direct the orientation of signal-anchor proteins: the topogenic role of the hydrophobic signal domain. J. Cell Biol. *137*, 555–562. https://doi.org/10.1083/jcb.137.3.555.
10. Chitwood, P.J., Juszkiewicz, S., Guna, A., Shao, S., and Hegde, R.S. (2018). EMC is required to initiate accurate membrane protein topogenesis. Cell *175*, 1507–1519.e16. https://doi.org/10.1016/j.cell.2018.10.009.
11. Higy, M., Junne, T., and Spiess, M. (2004). Topogenesis of membrane proteins at the endoplasmic reticulum. Biochemistry *43*, 12716–12722. https://doi.org/10.1021/bi048368m.
12. Itskanov, S., and Park, E. (2023). Mechanism of protein translocation by the Sec61 translocon complex. Cold Spring Harb. Perspect. Biol. *15*, a041250. https://doi.org/10.1101/cshperspect.a041250.
13. Van den Berg, B., Clemons, W.M., Collinson, I., Modis, Y., Hartmann, E., Harrison, S.C., and Rapoport, T.A. (2004). X-ray structure of a protein-conducting channel. Nature *427*, 36–44. https://doi.org/10.1038/nature02218.
14. Heinrich, S.U., Mothes, W., Brunner, J., and Rapoport, T.A. (2000). The Sec61p complex mediates the integration of a membrane protein by allowing lipid partitioning of the transmembrane domain. Cell *102*, 233–244. https://doi.org/10.1016/s0092-8674(00)00028-3.
15. High, S., Andersen, S.S., Görlich, D., Hartmann, E., Prehn, S., Rapoport, T.A., and Dobberstein, B. (1993). Sec61p is adjacent to nascent type I and type II signal-anchor proteins during their membrane insertion. J. Cell Biol. *121*, 743–750. https://doi.org/10.1083/jcb.121.4.743.
16. Martoglio, B., Hofmann, M.W., Brunner, J., and Dobberstein, B. (1995). The protein-conducting channel in the membrane of the endoplasmic reticulum is open laterally toward the lipid bilayer. Cell *81*, 207–214. https://doi.org/10.1016/0092-8674(95)90330-5.
17. O'Keefe, S., Zong, G., Duah, K.B., Andrews, L.E., Shi, W.Q., and High, S. (2021). An alternative pathway for membrane protein biogenesis at the endoplasmic reticulum. Commun. Biol. *4*, 828. https://doi.org/10.1038/s42003-021-02363-z.
18. Itskanov, S., Wang, L., Junne, T., Sherriff, R., Xiao, L., Blanchard, N., Shi, W.Q., Forsyth, C., Hoepfner, D., Spiess, M., and Park, E. (2022). A common mechanism of Sec61 translocon inhibition by small molecules. bioRxiv. https://doi.org/10.1101/2022.08.11.503542.
19. Baron, L., Paatero, A.O., Morel, J.D., Impens, F., Guenin-Macé, L., Saint-Auret, S., Blanchard, N., Dillmann, R., Niang, F., Pellegrini, S., et al. (2016). Mycolactone subverts immunity by selectively blocking the Sec61 translocon. J. Exp. Med. *213*, 2885–2896. https://doi.org/10.1084/jem.20160662.
20. McKenna, M., Simmonds, R.E., and High, S. (2017). Mycolactone reveals the substrate-driven complexity of Sec61-dependent transmembrane protein biogenesis. J. Cell Sci. *130*, 1307–1320. https://doi.org/10.1242/jcs.198655.
21. Tranter, D., Paatero, A.O., Kawaguchi, S., Kazemi, S., Serrill, J.D., Kellosalo, J., Vogel, W.K., Richter, U., Mattos, D.R., Wan, X., et al. (2020). Coibamide A targets Sec61 to prevent biogenesis of secretory and membrane proteins. ACS Chem. Biol. *15*, 2125–2136. https://doi.org/10.1021/acschembio.0c00325.
22. Chitwood, P.J., and Hegde, R.S. (2019). The role of EMC during membrane protein biogenesis. Trends Cell Biol. *29*, 371–384. https://doi.org/10.1016/j.tcb.2019.01.007.
23. Hegde, R.S. (2022). The function, structure, and origins of the ER membrane protein complex. Annu. Rev. Biochem. *91*, 651–678. https://doi.org/10.1146/annurev-biochem-032620-104553.
24. Wideman, J.G. (2015). The ubiquitous and ancient ER membrane protein complex (EMC): tether or not? F1000Res *4*, 624. https://doi.org/10.12688/f1000research.6944.2.
25. Anghel, S.A., McGilvray, P.T., Hegde, R.S., and Keenan, R.J. (2017). Identification of Oxa1 homologs operating in the eukaryotic endoplasmic reticulum. Cell Rep. *21*, 3708–3716. https://doi.org/10.1016/j.celrep.2017.12.006.
26. Lewis, A.J.O., and Hegde, R.S. (2021). A unified evolutionary origin for the ubiquitous protein transporters SecY and YidC. BMC Biol. *19*, 266. https://doi.org/10.1186/s12915-021-01171-5.
27. Hennon, S.W., Soman, R., Zhu, L., and Dalbey, R.E. (2015). YidC/Alb3/Oxa1 family of insertases. J. Biol. Chem. *290*, 14866–14874. https://doi.org/10.1074/jbc.R115.638171.
28. O'Donnell, J.P., Phillips, B.P., Yagita, Y., Juszkiewicz, S., Wagner, A., Malinverni, D., Keenan, R.J., Miller, E.A., and Hegde, R.S. (2020). The architecture of EMC reveals a path for membrane protein insertion. eLife *9*, e57887. https://doi.org/10.7554/eLife.57887.
29. Pfeffer, S., Burbaum, L., Unverdorben, P., Pech, M., Chen, Y., Zimmermann, R., Beckmann, R., and Förster, F. (2015). Structure of the native Sec61 protein-conducting channel. Nat. Commun. *6*, 8403. https://doi.org/10.1038/ncomms9403.
30. Guna, A., Volkmar, N., Christianson, J.C., and Hegde, R.S. (2018). The ER membrane protein complex is a transmembrane domain insertase. Science *359*, 470–473. https://doi.org/10.1126/science.aao3099.
31. Güngör, B., Flohr, T., Garg, S.G., and Herrmann, J.M. (2022). The ER membrane complex (EMC) can functionally replace the Oxa1 insertase in

mitochondria. PLoS Biol. *20*, e3001380. https://doi.org/10.1371/journal.pbio.3001380.

32. Hessa, T., Kim, H., Bihlmaier, K., Lundin, C., Boekel, J., Andersson, H., Nilsson, I., White, S.H., and von Heijne, G. (2005). Recognition of transmembrane helices by the endoplasmic reticulum translocon. Nature *433*, 377–381. https://doi.org/10.1038/nature03216.

33. Higy, M., Gander, S., and Spiess, M. (2005). Probing the environment of signal-anchor sequences during topogenesis in the endoplasmic reticulum. Biochemistry *44*, 2039–2047. https://doi.org/10.1021/bi047976z.

34. Volkmar, N., Thezenas, M.L., Louie, S.M., Juszkiewicz, S., Nomura, D.K., Hegde, R.S., Kessler, B.M., and Christianson, J.C. (2019). The ER membrane protein complex promotes biogenesis of sterol-related enzymes maintaining cholesterol homeostasis. J. Cell Sci. *132*, jcs223453. https://doi.org/10.1242/jcs.223453.

35. Devaraneni, P.K., Conti, B., Matsumura, Y., Yang, Z., Johnson, A.E., and Skach, W.R. (2011). Stepwise insertion and inversion of a type II signal anchor sequence in the ribosome-Sec61 translocon complex. Cell *146*, 134–147. https://doi.org/10.1016/j.cell.2011.06.004.

36. Párraga, G., Horvath, S.J., Eisen, A., Taylor, W.E., Hood, L., Young, E.T., and Klevit, R.E. (1988). Zinc-dependent structure of a single-finger domain of yeast ADR1. Science *241*, 1489–1492. https://doi.org/10.1126/science.3047872.

37. Marino, J., von Heijne, G., and Beckmann, R. (2016). Small protein domains fold inside the ribosome exit tunnel. FEBS Lett. *590*, 655–660. https://doi.org/10.1002/1873-3468.12098.

38. Conti, B.J., Elferich, J., Yang, Z., Shinde, U., and Skach, W.R. (2014). Cotranslational folding inhibits translocation from within the ribosome-Sec61 translocon complex. Nat. Struct. Mol. Biol. *21*, 228–235. https://doi.org/10.1038/nsmb.2779.

39. Bowers, P.M., Schaufler, L.E., and Klevit, R.E. (1999). A folding transition and novel zinc finger accessory domain in the transcription factor ADR1. Nat. Struct. Biol. *6*, 478–485. https://doi.org/10.1038/8283.

40. Goder, V., and Spiess, M. (2003). Molecular mechanism of signal sequence orientation in the endoplasmic reticulum. EMBO J. *22*, 3645–3653. https://doi.org/10.1093/emboj/cdg361.

41. Johnsson, N., and Varshavsky, A. (1994). Ubiquitin-assisted dissection of protein transport across membranes. EMBO J. *13*, 2686–2698. https://doi.org/10.1002/j.1460-2075.1994.tb06559.x.

42. Jungnickel, B., and Rapoport, T.A. (1995). A posttargeting signal sequence recognition event in the endoplasmic reticulum membrane. Cell *82*, 261–270. https://doi.org/10.1016/0092-8674(95)90313-5.

43. Liu, Z., Chen, O., Wall, J.B.J., Zheng, M., Zhou, Y., Wang, L., Vaseghi, H.R., Qian, L., and Liu, J. (2017). Systematic comparison of 2A peptides for cloning multi-genes in a polycistronic vector. Sci. Rep. *7*, 2193. https://doi.org/10.1038/s41598-017-02460-2.

44. Pleiner, T., Tomaleri, G.P., Januszyk, K., Inglis, A.J., Hazu, M., and Voorhees, R.M. (2020). Structural basis for membrane insertion by the human ER membrane protein complex. Science *369*, 433–436. https://doi.org/10.1126/science.abb5008.

45. Bai, L., You, Q., Feng, X., Kovach, A., and Li, H. (2020). Structure of the ER membrane complex, a transmembrane-domain insertase. Nature *584*, 475–478. https://doi.org/10.1038/s41586-020-2389-3.

46. Miller-Vedam, L.E., Bräuning, B., Popova, K.D., Schirle Oakdale, N.T., Bonnar, J.L., Prabu, J.R., Boydston, E.A., Sevillano, N., Shurtleff, M.J., Stroud, R.M., et al. (2020). Structural and mechanistic basis of the EMC-dependent biogenesis of distinct transmembrane clients. eLife *9*, e62611. https://doi.org/10.7554/eLife.62611.

47. Akopian, D., Shen, K., Zhang, X., and Shan, S.O. (2013). Signal recognition particle: an essential protein-targeting machine. Annu. Rev. Biochem. *82*, 693–721. https://doi.org/10.1146/annurev-biochem-072711-164732.

48. McKenna, M.J., Adams, B.M., Chu, V., Paulo, J.A., and Shao, S. (2022). ATP13A1 prevents ERAD of folding-competent mislocalized and misoriented proteins. Mol. Cell *82*, 4277–4289.e10. https://doi.org/10.1016/j.molcel.2022.09.035.

49. Kobayashi, K., Jomaa, A., Lee, J.H., Chandrasekar, S., Boehringer, D., Shan, S.O., and Ban, N. (2018). Structure of a prehandover mammalian ribosomal SRP·SRP receptor targeting complex. Science *360*, 323–327. https://doi.org/10.1126/science.aar7924.

50. Jumper, J., Evans, R., Pritzel, A., Green, T., Figurnov, M., Ronneberger, O., Tunyasuvunakool, K., Bates, R., Žídek, A., Potapenko, A., et al. (2021). Highly accurate protein structure prediction with AlphaFold. Nature *596*, 583–589. https://doi.org/10.1038/s41586-021-03819-2.

51. Voorhees, R.M., Fernández, I.S., Scheres, S.H.W., and Hegde, R.S. (2014). Structure of the mammalian ribosome-Sec61 complex to 3.4 Å resolution. Cell *157*, 1632–1643. https://doi.org/10.1016/j.cell.2014.05.024.

52. Ataide, S.F., Schmitz, N., Shen, K., Ke, A., Shan, S.O., Doudna, J.A., and Ban, N. (2011). The crystal structure of the signal recognition particle in complex with its receptor. Science *331*, 881–886. https://doi.org/10.1126/science.1196473.

53. Shen, K., Arslan, S., Akopian, D., Ha, T., and Shan, S.O. (2012). Activated GTPase movement on an RNA scaffold drives co-translational protein targeting. Nature *492*, 271–275. https://doi.org/10.1038/nature11726.

54. Nathan, H.C., Keith, C.C., Andreas-David, B., Kibeom, K., André, C.M., Preethi, R., Hirofumi, K., Laura, S., Jason, Y.L., Hera, C., James, Y.S.K., et al. (2022). OpenCell: endogenous tagging for the cartography of human cellular organization. Science *375*, 6585. https://doi.org/10.1126/science.abi6983.

55. White, S.H., and Wimley, W.C. (1999). Membrane protein folding and stability: physical principles. Annu. Rev. Biophys. Biomol. Struct. *28*, 319–365. https://doi.org/10.1146/annurev.biophys.28.1.319.

56. Pechmann, S., Chartron, J.W., and Frydman, J. (2014). Local slowdown of translation by nonoptimal codons promotes nascent-chain recognition by SRP in vivo. Nat. Struct. Mol. Biol. *21*, 1100–1105. https://doi.org/10.1038/nsmb.2919.

57. Bogdanov, M., Dowhan, W., and Vitrac, H. (2014). Lipids and topological rules governing membrane protein assembly. Biochim. Biophys. Acta *1843*, 1475–1488. https://doi.org/10.1016/j.bbamcr.2013.12.007.

58. Guna, A., Stevens, T.A., Inglis, A.J., Replogle, J.M., Esantsi, T.K., Muthukumar, G., Shaffer, K.C.L., Wang, M.L., Pogson, A.N., Jones, J.J., et al. (2022). MTCH2 is a mitochondrial outer membrane protein insertase. Science *378*, 317–322. https://doi.org/10.1126/science.add1856.

59. Petriman, N.A., Jauß, B., Hufnagel, A., Franz, L., Sachelaru, I., Drepper, F., Warscheid, B., and Koch, H.G. (2018). The interaction network of the YidC insertase with the SecYEG translocon, SRP and the SRP receptor FtsY. Sci. Rep. *8*, 578. https://doi.org/10.1038/s41598-017-19019-w.

60. Welte, T., Kudva, R., Kuhn, P., Sturm, L., Braig, D., Müller, M., Warscheid, B., Drepper, F., and Koch, H.G. (2012). Promiscuous targeting of polytopic membrane proteins to SecYEG or YidC by the Escherichia coli signal recognition particle. Mol. Biol. Cell *23*, 464–479. https://doi.org/10.1091/mbc.E11-07-0590.

61. McKenna, M.J., Sim, S.I., Ordureau, A., Wei, L., Harper, J.W., Shao, S., and Park, E. (2020). The endoplasmic reticulum P5A-ATPase is a transmembrane helix dislocase. Science *369*, eabc5809. https://doi.org/10.1126/science.abc5809.

62. Song, W., Raden, D., Mandon, E.C., and Gilmore, R. (2000). Role of Sec61alpha in the regulated transfer of the ribosome-nascent chain complex from the signal recognition particle to the translocation channel. Cell *100*, 333–343. https://doi.org/10.1016/s0092-8674(00)80669-8.

63. Fons, R.D., Bogert, B.A., and Hegde, R.S. (2003). Substrate-specific function of the translocon-associated protein complex during translocation across the ER membrane. J. Cell Biol. *160*, 529–539. https://doi.org/10.1083/jcb.200210095.

64. Sharma, A., Mariappan, M., Appathurai, S., and Hegde, R.S. (2010). In vitro dissection of protein translocation into the mammalian endoplasmic

reticulum. Methods Mol. Biol. *619*, 339–363. https://doi.org/10.1007/978-1-60327-412-8_20.

65. Schindelin, J., Arganda-Carreras, I., Frise, E., Kaynig, V., Longair, M., Pietzsch, T., Preibisch, S., Rueden, C., Saalfeld, S., Schmid, B., et al. (2012). Fiji: an open-source platform for biological-image analysis. Nat. Methods *9*, 676–682. https://doi.org/10.1038/nmeth.2019.

66. Görlich, D., and Rapoport, T.A. (1993). Protein translocation into proteoliposomes reconstituted from purified components of the endoplasmic reticulum membrane. Cell *75*, 615–630. https://doi.org/10.1016/0092-8674(93)90483-7.

67. Chandrasekaran, V., Juszkiewicz, S., Choi, J., Puglisi, J.D., Brown, A., Shao, S., Ramakrishnan, V., and Hegde, R.S. (2019). Mechanism of ribosome stalling during translation of a poly(A) tail. Nat. Struct. Mol. Biol. *26*, 1132–1140. https://doi.org/10.1038/s41594-019-0331-x.

68. Feng, Q., and Shao, S. (2018). In vitro reconstitution of translational arrest pathways. Methods *137*, 20–36. https://doi.org/10.1016/j.ymeth.2017.12.018.

STAR★METHODS

KEY RESOURCES TABLE

REAGENT or RESOURCE	SOURCE	IDENTIFIER
**Antibodies**		
EMC3/TM111 Recombinant Polyclonal Antibody	Invitrogen	711771; RRID: AB_2716909
SRP54 antibody	BD Biosciences	610940; RRID:AB_398253
Sec61α	Song et al.[62]	NA
Sec61β	Fons et al.[63]	NA
Peroxidase AffiniPure Goat Anti-Rabbit IgG (H+L)	Jackson ImmunoResearch	111-035-003 RRID: AB_2313567
**Chemicals, Peptides, and Recombinant Proteins**		
Bismaleimidohexane (BMH)	Thermo	22330
EasyTag L-[35S]-Methionine	Perkin Elmer	NEG709A001MC
m7G(5')ppp(5')G RNA Cap Structure Analog	New England Biolabs	S1404L
RNasin® Ribonuclease Inhibitor	Promega	N2515
Amino acid kit	Promega	L9961/L996B
SP6 Polymerase	NEB	M0207L
Creatine kinase	Roche	CK-RO, SKU# 10127566001
Creatine phosphate	Roche	CRPHO-RO, SKU# 10621714001
SuperSignal West Pico Chemiluminescent substrate	Thermo Fisher	34080
Rabbit Reticulocyte Lysate Mix	Sharma et al.[64]	N/A
DMEM, high glucose, GlutaMAX, pyruvate	Gibco	10569-010
PonceauS Solution	Sigma-Aldrich	P-7170-1L
TransIT 293	Mirus	MIR 2705
NEBuilder® HiFi DNA Assembly Master Mix	NEB	E2621L
Lipofectamine™ RNAiMAX transfection reagent	Invitrogen	13778075
Spermidine	Sigma	S0266
ATP	Roche	ATPDS-RO, SKU#10519979001
UTP	Sigma	U6875
CTP	Sigma	C1506
GTP	Roche	10106399001
GMPCPP	Jena Bioscience	NU-405S
Nuclease S7	Roche	10107921001
RNaseA	Thermo Scientific	EN0531
Benzonase	Millipore	E1014
SMPH (Succinimidyl 6-((beta-maleimidopropionamido)hexanoate))	Thermo Scientific	22363
ANTI-FLAG® M2 Affinity Gel	Millipore	A2220
CaptivA® Protein A Affinity Resin	CA-HF-0100	Repligen
Pierce™ Protein G Agarose	Thermo Scientific	20397
PNGase F	NEB	P0704S
SYPRO™ Ruby	Invitrogen	S12000
Proteinase K	Fisher	BP1700-100
**Experimental Models: Cell Lines**		
Flp-In 293 T-Rex Cells WT	Guna et al.[30]	N/A
Flp-In 293 T-Rex Cells ΔEMC6	Guna et al.[30]	N/A

(Continued on next page)

CellPress
OPEN ACCESS

Continued		
REAGENT or RESOURCE	SOURCE	IDENTIFIER
Oligonucleotides		
AGGTCTTGATACTCCTTGGTCGC GGTAGCGTAATCTGGAAC	IDT	SP64_R_099
TTCCAGATTACGCTACCGCGACC AAGGAGTATCAAGACCTTCAG	IDT	ASGR1_F_096
TAATGGTGATGGTGATGGTGAA GGAGAGGTGGCTCCTGGCT	IDT	ASGR1_R_097
GCCAGGAGCCACCTCTCCTTCACCA TCACCATCACCATTAATAAAACTCG	IDT	SP64_F_098
TCAACTTTGGCAGATCCACCATGG CGCATCACCATCAC	IDT	23L_F_193
TGGTGATGGTGATGCGCCATGGT GGATCTGCCAAAG	IDT	PC119_R_196
GAAGAAAACCCCGGTCCTGCCTA CCCATACGATGTTCCAGAT	IDT	PC119_F_195
GGAACATCGTATGGGTAGGCAGGA CCGGGGTTTTCTTCCACGTCTCCTG CTTGCTTTAACAGAGAGAAGTTCGT GGCGCCACTACCTCCGCCC	IDT	23L_R_194
AGCAGCAGGAAGTTGGTGGGC GTGTGAAGCGCTTTGAAGT	IDT	gb096_R_207
ACTTCAAAGCGCTTCACACGC CCACCAACTTCCTGCTGC	IDT	TMD2_F_206
ATGACAAGAGCGGCAGCGGC ATGCCTGGTCCGACCCC	IDT	gb096_104_F_209
CTGGGGGTCGGACCAGGCA TGCCGCTGCCGCTCTTG	IDT	gb104_R_208
GGCTACAATTAATACATAACC TTATGTATCATACACATACG	IDT	P2_TM1_012
CCCACCCCAAACGATctaTAA TAATAACTTAAGCATCAGCC GCTGCGTGCTCCCGA	IDT	66merLLL_R_001
CCCACCCCAAACGATctaTAA TAATAACTTAAGCATGGGG GTCGGACCAGGCAT	IDT	76merLLL_R_002
CCCACCCCAAACGATctaTA ATAATAACTTAAGCATGCGC CCTGAGGATCCCACG	IDT	86merLLL_R_003
CCCACCCCAAACGATctaTAA TAATAACTTAAGCATCGCC CGGGCGGCC	IDT	96merLLL13_011
CCCACCCCAAACGATctaTAA TAATAACTTAAGCATATTTTT CCTCTGCCGGACAG	IDT	106merLLL_005
CCCACCCCAAACGATctaTAA TAATAACTTAAGCATGCGG CCTGCACTC	IDT	116merLLL14_013
CCCACCCCAAACGATctaTAA TAATAACTTAAGCATCCACA TCCCCCCGG	IDT	126merLLL14_014
CCCACCCCAAACGATctaTAA TAATAACTTAAGCATGGTAC CGCCAACTTTGAGC	IDT	141merLLL_R_008

(*Continued on next page*)

Continued

REAGENT or RESOURCE	SOURCE	IDENTIFIER
CCCACCCCAAACGATctaTAA TAATAACTTAAGCATGTGGA GCAGCAGGCTGG	IDT	ASGR1FL_70pTMD_R_108
CCCACCCCAAACGATctaTA ATAATAACTTAAGCATCATCT GGCTGCTCAGGCTCCGCAGG TCAGACACGAACTGCTTC ACGTGGAGCAGCAGGCTGG	IDT	ASGR1FL _85pTMD_230
CCCACCCCAAACGATctaTAA TAATAACTTAAGCATCCAA GTGGACTGTCCTTTGAGG	IDT	TMEM97_70pTMD_R_163
CCCACCCCAAACGATctaTA ATAATAACTTAAGCATCAC CGTGGCCCCCAC	IDT	AQP6_70pTMD_R_164
Recombinant DNA		
SP64 HA-TAAR5 TM1-β-6His	Chitwood et al.[10]	PC119
pOG44	Invitrogen	V600520
SP64 HA-ASGR1(FL)-His	This study	HW040
SP64 HA-glyc-TAAR5(N+3)-His	This study	HW125
SP64 HA-glyc-TAAR5(N-3)-His	This study	HW126
SP64 His-23L-mEGFP-P2A-TAAR5TM1-3	This study	HW101
pcDNA5FRTΔTO-EMC3-3xFlag	This study	HW009
pcDNA5FRTΔTO-EMC3 E63K/D213K/E223K-3xFlag	This study	HW016
pcDNA5FRTΔTO-EMC3 F148L-3xFlag	This study	HW018
pcDNA5FRTΔTO-EMC3 R13E-3xFlag	This study	HW019
pcDNA5FRTΔTO-EMC3 R13C-3xFlag	This study	HW069
pcDNA5FRTΔTO-EMC3 R31C-3xFlag	This study	HW070
pcDNA5FRTΔTO-EMC3 E63C-3xFlag	This study	HW071
pcDNA5FRTΔTO-EMC3 M101C-3xFlag	This study	HW072
pcDNA5FRTΔTO-EMC3 N114C-3xFlag	This study	HW073
pcDNA5FRTΔTO-EMC3 F148C-3xFlag	This study	HW074
pcDNA5FRTΔTO-EMC3 R180C-3xFlag	This study	HW075
pcDNA5FRTΔTO-EMC3 E223C-3xFlag	This study	HW076
pcDNA5FRTΔTO-EMC3 E240C-3xFlag	This study	HW077
pcDNA5FRTΔTO-EMC3 S259C-3xFlag	This study	HW078
pcDNA5FRTΔTO-EMC3 EMC3 R31E/R180E-3xFlag	This study	HW117
pcDNA5FRTΔTO-EMC3 EMC3 R31L/R180L-3xFlag	This study	HW085
SP64 TAAR5 126mer Y3C	This study	HWgb001
SP64 TAAR5 126mer P8C	This study	HWgb002
SP64 TAAR5 126mer S18C	This study	HWgb003
SP64 TAAR5 126mer H28C	This study	HWgb004
SP64 TAAR5 126mer L38C	This study	HWgb005
SP64 TAAR5 126mer V48C	This study	HWgb006
SP64 TAAR5 126mer V58C	This study	HWgb007
SP64 ASGR1_70pTMD	This study	HWgb010
TMEM97_70pTMD	This study	HWgb012
AQP6_70pTMD	This study	HWgb013
SP64 TAAR5 126mer S18C N0	This study	HWgb014
SP64 TAAR5 126mer S18C N-1	This study	HWgb015
SP64 TAAR5 126mer S18C C0	This study	HWgb017
SP64 TAAR5 126mer S18C C-1	This study	HWgb018

(Continued on next page)

Continued

REAGENT or RESOURCE	SOURCE	IDENTIFIER
SP64 HA-ASGR1-ZNF-74	This study	HWgb099
SP64 HA-ASGR1- ZNF-79	This study	HWgb100
SP64 HA-ASGR1- ZNF-84	This study	HWgb101
SP64 HA-ASGR1- ZNF-89	This study	HWgb102
SP64 HA-ASGR1- ZNF-224	This study	HWgb103
SP64 TAAR5 TM1-3	This study	HWgb104
Software and Algorithms		
Adobe Illustrator	Adobe	https://www.adobe.com/uk/creativecloud.html
Fiji	Schindelin et al. [65]	https://fiji.sc/
GraphPad Prism 8	GraphPad	www.graphpad.com

RESOURCE AVAILABILITY

Lead contact

Further information and requests for resources and reagents should be directed to and will be fulfilled by the lead contact, Ramanujan S. Hegde (rhegde@mrc-lmb.cam.ac.uk).

Materials availability

Plasmids generated in this study will be available upon request.

Data and code availability

This study did not analyze any datasets.

This paper does not report original code.

Any additional information required to reanalyze the data reported in this paper is available from the lead contact upon request.

EXPERIMENTAL MODEL AND SUBJECT DETAILS

Cell lines

WT and ΔEMC6 Flp-In™ T-REx™ 293 cells were cultured in DMEM, high glucose, GlutaMAX™ Supplement, pyruvate (Gibco 10569-010) containing 10% FBS are reported and characterized before.[30] Cells were cultured at 37°C with 5% CO_2. All cell lines are female, routinely verified for the presence and absence of EMC subunits and not authenticated further.

METHOD DETAILS

DNA

All plasmids used in this study are verified by sequencing. Mammalian WT EMC3 expression construct is in pcDNA5FRTΔTO-3xFlag backbone and is described before.[28] Point mutations of EMC3 constructs were generated by site-directed mutagenesis and are listed in the key resource table.

Plasmids for in vitro transcription and translation are in an SP64 backbone containing SP6 promoter. N_{exo} G protein-coupled receptor TAAR5 signal-anchor (SA) reporter cassettes were described before.[10] Point mutations of TAAR5 SA reporter were generated by site-directed mutagenesis and are listed in the key resource table. HA-ASGR1 was generated by Gibson assembly following manufacturer's protocol (NEB, E2621L). ASGR1 was PCR amplified with oligos HWO96+97; SP64 backbone was linearized by HWO98+99. 23L-P2A-TAAR5TMD1-3 was generated by a two-step assembly. First 23L-P2A (amplified by HWO193+194) was inserted in front of HA-TAAR5-SA/TMD1 (linearized by HWO195+196); then TAAR5TMD2-3 (amplified by HWO206+208) was inserted after 23L-P2A-TAAR5-TMD1 (linearized by HWO207+209). Oligo sequences are listed in the key resource table. All other DNA are ordered as gBlocks from IDT and are listed in the key resource table.

Generation of stable cell lines

To generate stable cell lines expressing WT or mutant EMC3, Flp-In™ system was used following manufactures' protocol (Invitrogen). Briefly, Flp-In™ 293 T-REx cell lines were plated in 6-well plates for 16hrs. Two separate 250 μL transfection mixes were assembled in Opti-MEM (Invitrogen, 31985-088): i). 200ng of pcDNA5FRTΔTO:EMC3-TEV-3xFLAG (WT or mutants) together with 1800ng of pOG44 (encoding the Flp recombinase); ii). 6 μL of TransIT®-293 Transfection Reagent (Mirus MIR 2700). After 48-72hrs, cells

were split into 10cm plates with mediate containing 100 μg/ml hygromycin B (selecting for cells had undergone Flp-mediated recombination). After two weeks of selection and expansion, the whole population of stable cells were used for downstream analysis.

Knockdown with siRNA

For RNAi experiments, cells were transfected with Silencer™ Select Negative Control No. 1 siRNA (Invitrogen, 4390843) or 12.5nM siRNA against SEC61A1 (Ambion s26721). Cells were plated for 16hrs the day before transfection. siRNAs were transfected using Lipofectamine™ RNAiMAX transfection reagent (Invitrogen 13778075) for 16hrs. 24hrs after the first transfection ended, same concentrations of siRNAs were transfected with the same protocol. Cells were harvested 16hrs after the second transfection for further analysis.

Preparation of semi-permeabilized cells

90-100% confluent cells were harvested by trypsinization and pelleting. Cell pellets were washed once with 1xPBS and resuspended in 1xRNC buffer [50 mM HEPES pH 7.4, 100 mM KOAc, 5 mM $Mg(OAc)_2$] containing 0.01% purified digitonin.[66] Cell suspension was incubated on ice for 10min for permeabilization and pelleted. Semi-permeabilized cell pellets were washed once with 1xRNC and either resuspended in 0.5xRNC buffer at concentration of 60000-10000 cells/μL for downstream experiments or treated with nuclease.

In co-translational experiments, SPCs were further digested by nuclease S7 to avoid interference of endogenous mRNA. SPCs were resuspended in 100μL 1xRNC containing 1mM $CaCl_2$ and 150 units/ml Nuclease S7 (Roche 10107921001). Nuclease digestion was 10min on ice and terminated by adding final concentration of 2mM EGTA. Nuclease digested cells were pelleted and washed once with 1XRNC buffer and resuspended in 0.5xRNC buffer at concentration of 60000-10000 cells/μL.

In vitro transcription and translation

Homemade SP6 promoter-mediated transcription and translation systems were described before.[64] Briefly, transcription was at 37°C for 1hr in a reaction containing the following components: 5-10ng/μL purified DNA (PCR products purified with Qiagen PCR Purification Kit, 28104); and 0.4 U/μl SP6 RNA polymerase (NEB M0207L); 0.8 U/μl RNasin® Ribonuclease Inhibitor (Promega, N2515/N251B); 40 mM HEPES, pH 7.4; 6 mM $MgCl_2$; 2 mM spermidine (Sigma S0266); 10 mM reduced glutathione; 0.5 mM ATP (Roche, ATPDS-RO, SKU#10519979001); 0.5 mM UTP (Sigma, U6875); 0.5 mM CTP (Sigma, C1506); 0.1 mM GTP (Roche, 10106399001); 0.33 mM m7G(5')ppp(5')G RNA Cap Structure Analog (NEB, S1404L).

Translation was at 32°C for 15-30min containing the following components: 5% volume of transcription; 34% volume nuclease-treated crude rabbit reticulocyte lysate (Green Hectares); 10% volume of membrane source (SPCs unless otherwise noted); 20mM HEPES, pH=7.4; 50mM potassium acetate; 2mM magnesium acetate; 12mM creatine phosphate (Roche, CRPHO-RO, SKU# 10621714001); 1mM ATP; 1mM GTP; 1mM reduced glutathione; 0.3mM spermidine (Sigma S0266); 0.04mg/mL creatine kinase (Roche, CK-RO, SKU# 10127566001); 0.05mg/mL tRNA (purified from pig liver); 40μM each of the 19 amino acid except for Methionine (Promega, L9961/L996B); 0.5 μCi/μl EasyTag™ L-[35S]-Methionine (PerkinElmer, NEG709A001MC). Where indicated, Apratoxin A was added to the reaction at final concentration of 2μM. In experiments containing ADR1a, 0.2mM Zn^{2+} was included in translation where indicated. In experiments with 23L-P2A-TMD1-3, cRM was used instead of SPCs. cRM preparation was described before (Walter and Blobel,1983).

Isolation of ribosome nascent chain complexes (RNCs)

RNCs of distinct lengths are isolated from translation reactions stalled at defined positions. Stalling was achieved by programing three consecutive leucine residues (UUA) into the mRNA (primers are listed in supplemental table). Translation was performed as before omitting membrane source and pig liver tRNA (only endogenous tRNAs from RRL was used). Endogenous RRL tRNA severely lacks tRNAs to decode UUA codon,[67,68] which result in translation to stall. 200μL of translation reaction was overlaid to a 2mL 10-50% sucrose gradient, which contains five equal fractions of 10%, 20%, 30%, 40% and 50% sucrose (top to bottom) in 1xRNC buffer. Gradients were centrifuged for 1hr at 4°C in a TLS-55 swinging-bucket rotor in a Beckman Coulter Optima MAX-XP Ultracentrifuge with the slowest acceleration and deceleration. Eleven 200 μL fractions were collected from the top and ribosome/RNC fractions 6-8 were pulled and mixed with final 1mM GTP for downstream analysis. In experiments stalling RNCs on SRP receptor at the ER membrane, GTP is either omitted or slowly-hydrolyzed GTP analog GMPCPP (Jena Bioscience NU-405S) is added to final of 0.1mM concentration.

In vitro insertion assays with purified RNCs

10μL of purified RNCs were incubated with 1μL of SPCs of desired genotype at 32°C for 10min. Where indicated, Apratoxin A was added to the reaction at final concentration of 2μM. Cells were then returned to ice and all following steps are performed at 4°C. Cells were pelleted and supernatant removed by aspiration. Cell pellets were resuspended in 10μL Tris/solA [100mM Tris pH=8; 50μg/mL RNaseA (Thermo Scientific EN0531); 0.05%SDS; 10mM EDTA; 0.25U/μL benzonase (Millipore E1014)] and incubated for 10-20min to release nascent chains from ribosomes. Reactions were terminated by adding 10uL of 5x sample buffer and one fourth of the total volume was analyzed on SDS-PAGE.

Protease protection assays with purified RNCs

100μL of purified ASGR1-SA+70 RNCs were mixed with 10μL of WT SPCs at 32°C for 10min. Cells were pelleted, supernatant removed by aspiration and returned to ice. Cell pellets were resuspended in 30μL 1xRNC buffer and divided in half. One fifth (6μL) left untreated, pelleted and resuspended in 20μL Tris/solA and incubated for 10-20min and analyzed as total products after mixing with 20uL of 5x sample buffer. The rest (24μL) were treated with final concentration of 0.5mg/mL Proteinase K (Fisher BP1700-100) for 50min, which was then quenched by 5mM PMSF for 2-5min. The entire reaction was transferred to 66μL of boiling 1% SDS, 100 mM Tris-Cl, pH 8.0 and boiled to denature for 10min. One fifth (18μL) was mixed with 2μL of 10xTris/solA for 10-20min at room temperature, and analyzed after mixing with 20uL of 5x sample buffer.

Site-specific chemical crosslinking

40μL of purified RNCs were incubated with 4μL of SPCs at 32°C for 10min. Cells were then returned to ice and all following steps are performed at 4°C. Cells were pelleted and supernatant removed by aspiration. Cell pellets were resuspended in 40μL 1xRNC buffer and divided in half. One half (20μL) left untreated, pelleted and resuspended in 10μL Tris/solA and incubated for 10-20min. One half (20μL) were treated with final concentration of 200μM of SMPH (Thermo Scientific 22363) for 30min or 250μM of BMH (Thermo Scientific 22330) for 10min. Crosslinking reactions were quenched with 50mM Tris pH=7.4; 5mM DTT (for SMPH), or 25mM DTT (for BMH). Cells were then pelleted resuspended in 10μL Tris/solA and incubated for 10-20min. Both untreated and crosslinked samples were mixed with 10uL of 5x sample buffer and analyzed on SDS-PAGE.

Immunoprecipitation

Crosslinking reactions were scaled up 10 times and performed as described before. Crosslinked materials were denatured in 50-100 μL of buffer containing final 1% SDS and 100mM Tris pH=8 and boiled for 10min. Denatured materials were subjected to immunoprecipitation by mixing with 2.5μL of anti-FLAG® M2 affinity gel (Millipore A2220), protein A (Repligen CA-HF-0100) or protein G agarose beads (Thermo Scientific 20397) that is washed and resuspended in 1mL of denaturing IP buffer (1xPBS, 250mM NaCl, 0.5% TX-100, 10mM Imidazole). Protein A beads was mixed with 1.5μL of Sec61β antibody; protein G was mixed with 5μL of SRP54 antibody (BD Biosciences 610940). Mixture was rotated end-over-end for 1.5hrs (for FLAG) or 3hrs (for protein A/G). Beads were washed twice with denaturing IP buffer and eluted by boiling in 10μL of 2.5x sample buffer for 10min.

In deglycosylation experiments, crosslinked samples were split into two halves after denaturation with 0.5% SDS and 50mM Tris pH=8. One half left untreated and the other half were mixed with final of 1% NP-40, 1x GlycoBuffer 2 and 25U/μL of PNGase F (NEB, P0704S) and digested at 32°C for 30min. Both haves were subjected to immunoprecipitation as described before.

In native IP experiments, crosslinked materials were resuspended in native IP buffer [50mM HEPES, pH=7.4, 200mM NaCl, 2mM $Mg(OAc)_2$, 1% TX-100] and incubated on ice for 10min. Solubilized membranes were cleared by 10min spin at max speed at 4°C. Supernatant was subject to native IP with 2.5μL of anti-FLAG® M2 affinity gel. Mixture was rotated end-over-end for 1.5hrs. Beads were washed four times with lysis buffer and transferred to a new tube after the last wash and then eluted by boiling in 10μL of 2.5x sample buffer for 10min.

Affinity purification of EMC

Confluent 10cm dishes stably expressing WT or mutant FLAG tagged EMC3 were harvested with trypsin and centrifugation. Cells were resuspend cells in 1.2mL lysis buffer [50mM HEPES, pH=7.4, 200mM NaCl, 2mM $Mg(OAc)_2$, 1% TX-100] and kept on ice for 10min. Cell lysate was centrifuged at 15k rpm for 10min at 4°C. Supernatant (~1.1mL) was transferred into a new tube. 40μL of lysate was saved and mixed with 20uL of 5x sample buffer as input/lysate. 1mL of lysate was transferred to a new tube and mixed with 15μL of anti-FLAG® M2 affinity gel (equilibrated with lysis buffer). Mixture was rotated end-over-end for 1.5hrs. Beads were washed four times with lysis buffer and transferred to a new tube after the last wash. Purified EMC was eluted by shaking beads at room temperature at 400rpm in a thermomixer in 20μL in lysis buffer containing of 0.25mg/mL 3xFLAG peptide. Elution was mixed with 20μL of 5x sample buffer for SDS-PAGE and SYPRO™ Ruby (Invitrogen S12000) staining according to manufacturer's protocol.

SDS-PAGE and western blotting

Total cell lysate was denatured in 2.5x sample buffer and adjusted to same concentration. Cell lysates were separated by Tris-Tricine SDS-PAGE and were transferred to 0.2 μm nitrocellulose membranes. Membranes were stained with Ponceau S to monitor loading and blocked in 5% dry milk dissolved in PBST (0.1% Tween 20) at room temperature for 1hr. Blocked membrane was incubated with rabbit EMC3 antibody (Invitrogen, 711771) or rabbit Sec61α antibody (homemade) at 1:5000 overnight or at room temperature for 1hr. Blots were washed in PBST for 20min and incubated with Peroxidase AffiniPure Goat Anti-Rabbit IgG (H+L) (Jackson ImmunoResearch 111-035-003) for 1-2hrs. After washing in PBST for 20min, blots were developed with SuperSignal™ West Pico PLUS Chemiluminescent Substrate (Thermo Scientific, 34580).

QUANTIFICATION AND STATISTICAL ANALYSIS

Quantification of autoradiographs

Background-subtracted band intensities from phosphor screens were quantified in Fiji.

Statistics

All statistical analyses were performed in GraphPad Prism. One-way ANOVA tests were performed and p values were derived from Tukey's test. ns=not significant, ***$p<0.001$.

Reproducibility

Reproducibility and reliability of the findings has been ensured because all experiments were performed on separate and fully independent occasions and verified to give the same result as the example shown in the figure.

Molecular Cell, Volume *83*

Supplemental information

Mechanism of signal-anchor triage

during early steps of membrane protein insertion

Haoxi Wu and Ramanujan S. Hegde

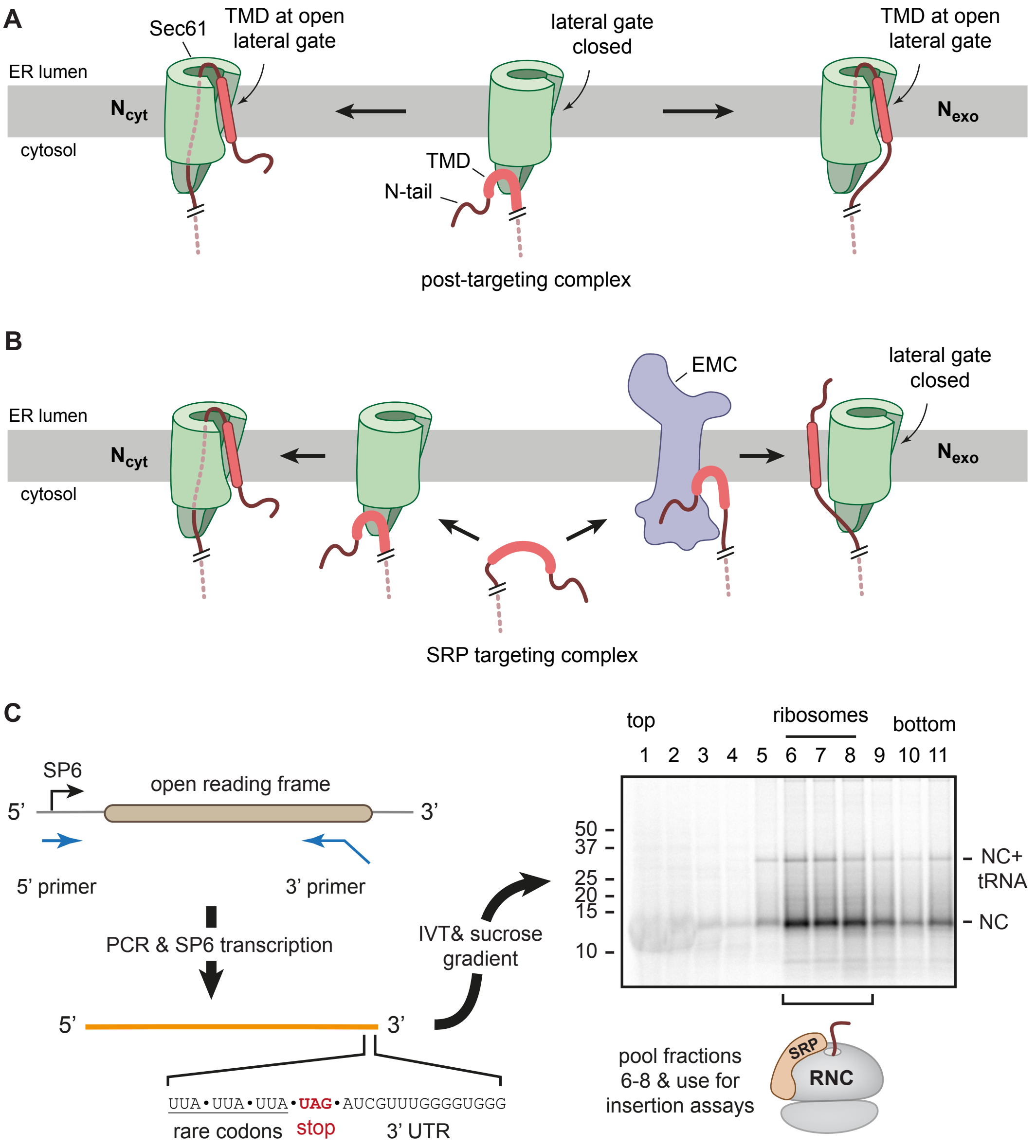

Figure S1. Models of membrane protein topogenesis, related to Figure 1

(A) Model for Sec61-mediated SA topogenesis based on Higy et al., 2004 [S1]. After targeting to the membrane, RNCs dock at the Sec61 complex. The SA is inserted in either the N_{exo} or N_{cyt} orientation. In both cases, the SA passes through Sec61's lateral gate, with one of the SA's flanking domains translocating through Sec61's central channel.

(B) Model for SA topogenesis based on Chitwood and Hegde, 2019 [S2]. After targeting to the membrane, N_{exo} insertion is mediated by the EMC insertase, after which the ribosome docks at the Sec61 complex. N_{cyt} insertion is mediated by the Sec61 complex.

(C) Method to produce RNCs stalled at a defined site by in vitro translation. The template for transcription is generated by PCR with a 3' primer that encodes three consecutive rare codons (TTA), a stop codon and a 3'UTR sequence. The PCR product is transcribed in vitro and translated in RRL containing only its endogenous tRNAs. RRL contains limited amounts of the tRNA used to decode the UUA codon [S3, S4], causing stalling at the three UUA codons. The translation reaction is separated by centrifugation through a 10-50% sucrose gradient and the RNCs are collected for downstream assays. A representative example (TAAR5-SA+70) is shown on the right after SDS-PAGE and autoradiography. Intact RNCs co-fractionate with ribosomes in fractions 6-8. Note that the peptidyl-tRNA bond on most nascent chains is hydrolyzed during electrophoresis at basic pH, but is stable at neutral pH.

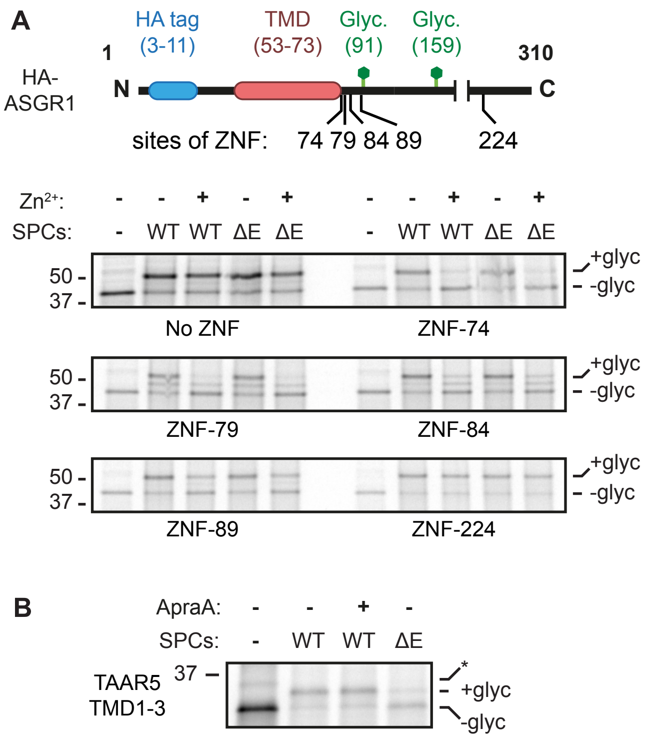

Figure S2. EMC-dependent SA insertion requires late targeting, related to Figure 2

(A) Top: diagram of HA-ASGR1 constructs without or with a zinc finger from ADR1 (ZNF) inserted immediately at the indicated positions. Bottom: ^{35}S-methionine labeled HA-ASGR1 constructs lacking or containing ZNF were translated in the absence or presence of SPCs from wild type (WT) or ΔEMC (ΔE) HEK293 cells without or with Zn^{2+} where indicated. After translation, the SPCs were recovered by centrifugation and analyzed by SDS-PAGE and autoradiography. An aliquot of the sample lacking SPCs was analyzed directly. The glycosylated (+glyc.) and non-glycosylated (-glyc.) products are indicated. Panels of ZNF, ZNF-74, ZNF-224 are reproduced from Fig. 2A for comparison.

(B) TAAR5(TMD1-3) is EMC-dependent. ^{35}S-methionine labeled TAAR5(TMD1-3) was translated in the absence or presence of SPCs derived from wild type (WT) or ΔEMC (ΔE) HEK293 cells. The Sec61 lateral gate inhibitor ApraA was included where indicated. After translation, the SPCs were recovered by centrifugation and analyzed by SDS-PAGE and autoradiography. An aliquot of the sample lacking SPCs was analyzed directly. The glycosylated (+glyc.) and non-glycosylated (-glyc.) products are indicated. Asterisk indicates a product seen in samples lacking SPCs, and may represent ubiquitin-modified substrate.

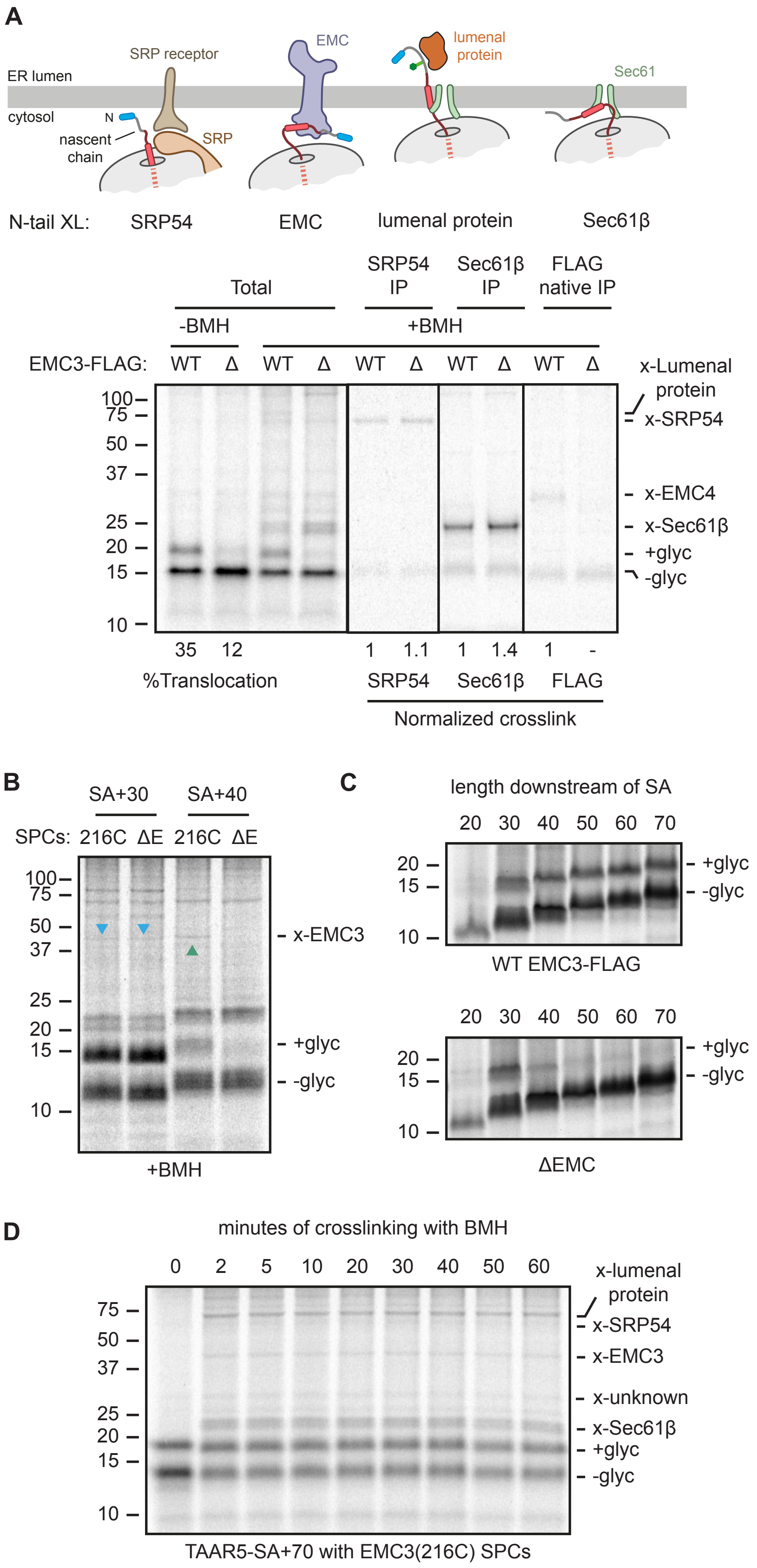
A
ER lumen
cytosol
SRP receptor
EMC
lumenal protein
Sec61
N
nascent chain
SRP
N-tail XL: SRP54 EMC lumenal protein Sec61β
Total
SRP54 IP
Sec61β IP
FLAG native IP
-BMH
+BMH
EMC3-FLAG: WT Δ WT Δ WT Δ WT Δ WT Δ
x-Lumenal protein
x-SRP54
x-EMC4
x-Sec61β
+glyc
-glyc
35 12
%Translocation
1 1.1 1 1.4 1 -
SRP54 Sec61β FLAG
Normalized crosslink
B
SA+30 SA+40
SPCs: 216C ΔE 216C ΔE
x-EMC3
+glyc
-glyc
+BMH
C
length downstream of SA
20 30 40 50 60 70
+glyc
-glyc
WT EMC3-FLAG
ΔEMC
D
minutes of crosslinking with BMH
0 2 5 10 20 30 40 50 60
x-lumenal protein
x-SRP54
x-EMC3
x-unknown
x-Sec61β
+glyc
-glyc
TAAR5-SA+70 with EMC3(216C) SPCs

Figure S3. Detection of EMC-substrate interaction, related to Figure 3

(A) Top: diagram showing putative factors sampled by the N-tail of an SA-containing RNC. Bottom: ^{35}S-methionine labeled TAAR5-SA+70 RNCs were incubated with SPCs containing EMC3(WT)-FLAG or lacking EMC (ΔE). One aliquot was analyzed directly (-BMH) and another treated with BMH, a sulfhydryl-reactive homo-bifunctional crosslinker (+BMH). The crosslinked products were analyzed directly, after denaturing IP with indicated antibodies or after native IP of EMC3 via the FLAG tag. The non-glycosylated (-glyc) and glycosylated (+glyc) products and crosslinks to Sec61β, EMC4, SRP54 and an ER lumenal protein are indicated. Prior to SDS-PAGE, tRNA was digested from the nascent chain using RNase A.

(B) Crosslinking analysis of TAAR5-SA+30 and TAAR5-SA+40 RNCs. ^{35}S-methionine labeled RNCs of TAAR5-SA+30 and TAAR5-SA+40 were incubated with SPCs containing EMC3(216C)-FLAG or lacking EMC (ΔE), crosslinked with BMH, and analyzed directly. The EMC3 crosslink is indicated with an upward green arrow. In TAAR5-SA+30 samples, a crosslink product of unknown identity with similar size to EMC3 is marked with downward blue arrows. This product remains in ΔEMC SPCs.

(C) Insertion assays for RNCs of various lengths and their dependence on EMC. ^{35}S-methionine labeled TAAR5 RNCs of indicated length were incubated with WT or ΔEMC (ΔE) SPCs. The glycosylated (+glyc) and non-glycosylated (-glyc) products are indicated. Note that prior to SDS-PAGE, tRNA was digested from the nascent chain using RNase A.

(D) Time course of BMH crosslinking. ^{35}S-methionine labeled TAAR5-SA+70 RNCs were incubated with EMC3(216C)-FLAG SPCs, crosslinked with BMH for the indicated duration, and analyzed directly.

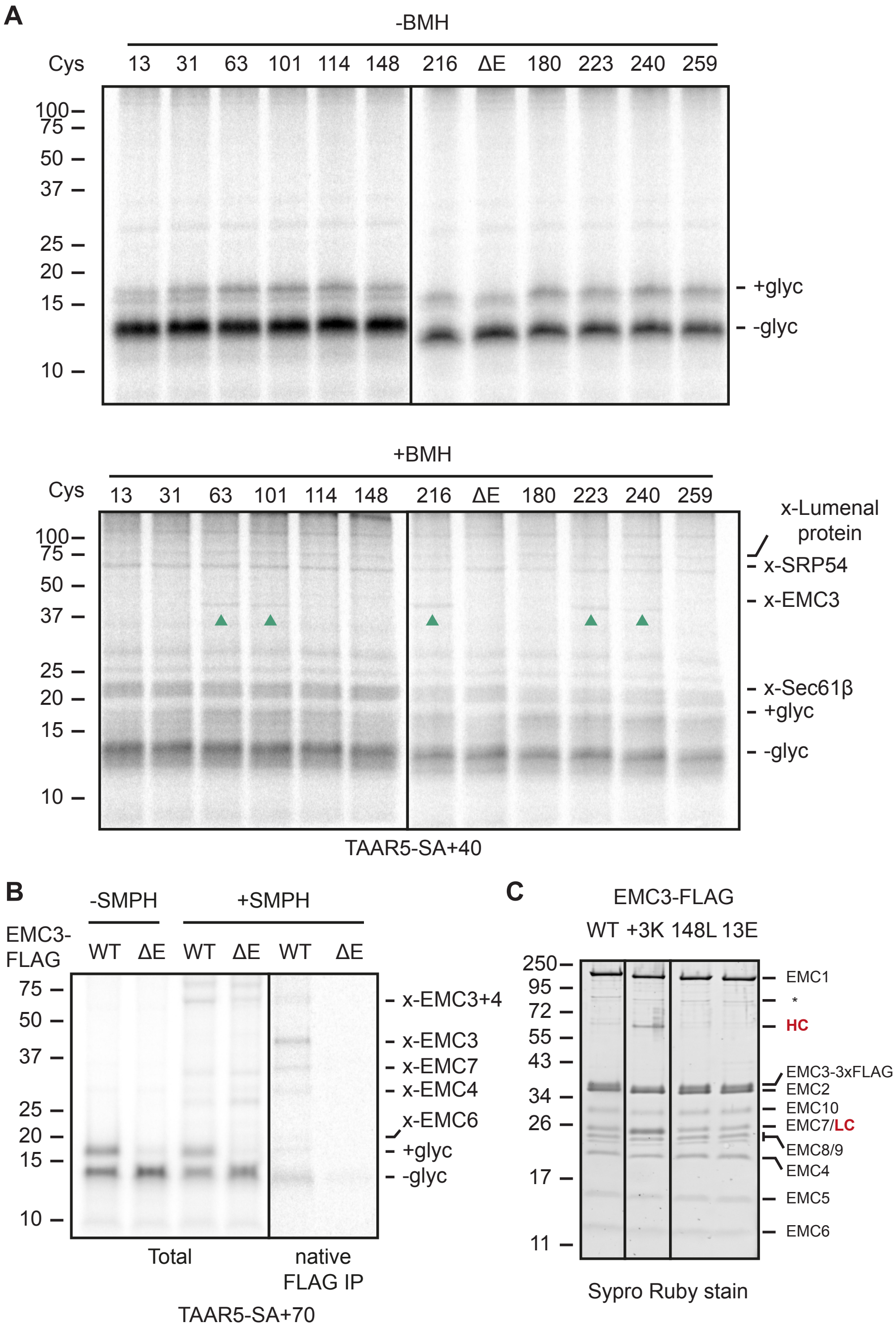

Figure S4. Mapping of EMC-substrate interactions, related to Figure 4

(A) Mapping substrate interaction with single cysteine variants of EMC3. ^{35}S-methionine labeled TAAR5-SA+40 RNCs were incubated with indicated EMC3-FLAG single cysteine variant SPCs, crosslinked with BMH and analyzed directly. The non-glycosylated (-glyc) and glycosylated (+glyc) products and crosslinks to Sec61β, EMC3 (upward green arrows), SRP54 and an ER lumenal protein are indicated. Prior to SDS-PAGE, tRNA was digested from the nascent chain using RNase A.

(B) Crosslinking reactions between ^{35}S-methionine labeled TAAR5-SA+70 RNCs and SPCs stably expressing EMC3-FLAG (WT) versus SPCs lacking EMC (ΔE). Crosslinking was with the sulfhydryl-amine heterobifunctional crosslinker SMPH. Samples were analyzed directly (total) or after native IP of EMC3 via the FLAG tag. The non-glycosylated (-glyc) and glycosylated (+glyc) products and EMC crosslinks consistent with EMC4, EMC6 and EMC7 are indicated.

(C) EMC3 mutations do not affect EMC integrity or abundance. Cells continuously expressing EMC3-FLAG (WT or indicated mutations) were harvested and EMC was affinity purified under native conditions via the FLAG tag. The products eluted from the affinity resin using FLAG peptide were analyzed by SDS-PAGE and Sypro-Ruby staining. Note that all EMC subunits are recovered in identical abundances and ratios for each of the mutants compared to the WT. A small amount of IgG heavy chain (HC) and light chain (LC) contaminate the +3K sample, and are indicated. The samples are all from the same experiment analyzed on the same gel, with vertical lines indicating places where intervening lanes of other samples were removed.

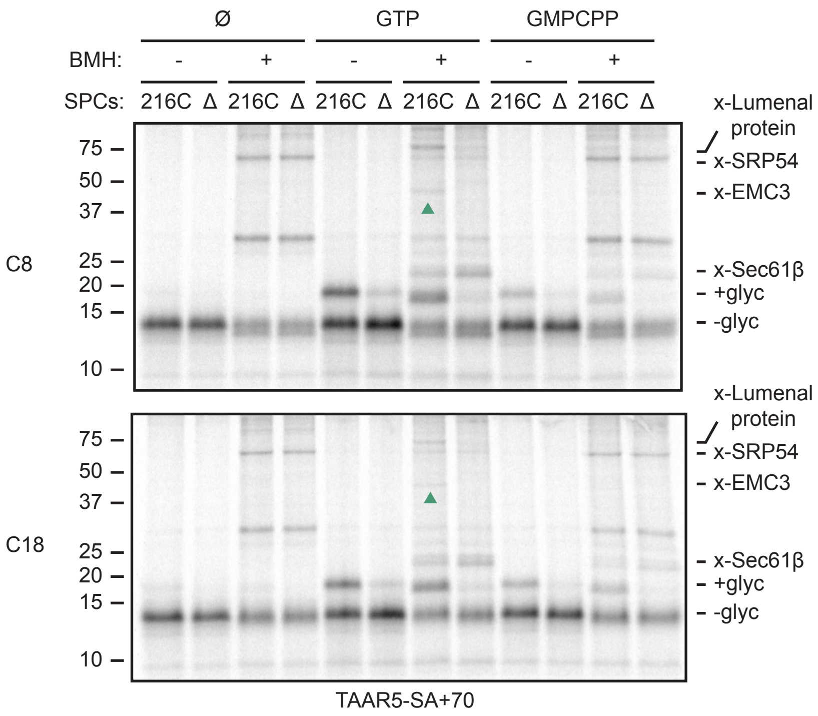

Figure S5. SRP release is required for EMC-substrate interaction, related to Figure 5

^{35}S-methionine labeled TAAR5-SA+70 RNCs with a cysteine at position 8 (top) or 18 (bottom) were mixed with GTP, the slowly-hydrolyzed GTP analog GMPCPP or nothing (Ø), then incubated with SPCs containing EMC3(216C)-FLAG or lacking EMC (Δ). Pelleted SPCs were subject to BMH crosslinking as indicated and analyzed directly. The non-glycosylated (-glyc) and glycosylated (+glyc) products and crosslinks to Sec61β, EMC3 (upward green arrows), SRP54 and a lumenal protein are indicated.

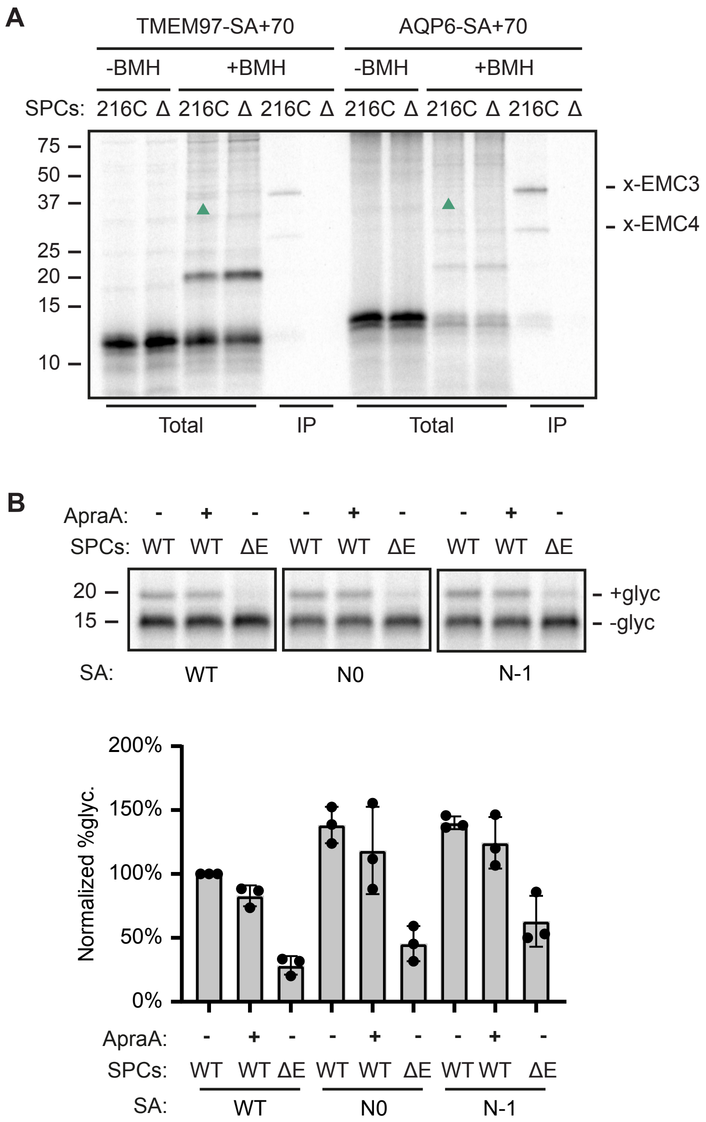

Figure S6. Determinants for EMC-substrate interaction, related to Figure 6

(A) ^{35}S-methionine labeled RNCs of TMEM97-SA+70 or AQP6-SA+70 were incubated with SPCs stably expressing EMC3(216C)-FLAG or lacking EMC (ΔE). One aliquot was analyzed directly (-BMH) and another treated with BMH (+BMH). The crosslinked products were analyzed directly, or after native IP of EMC3 via the FLAG tag. The non-glycosylated (-glyc) and glycosylated (+glyc) products and crosslinks to EMC3 (upward green arrows) and EMC4 are indicated. Prior to SDS-PAGE, tRNA was digested from the nascent chain using RNase A.

(B) ^{35}S-methionine labeled RNCs of the indicated TAAR5-SA+70 variants were incubated with SPCs stably expressing either wild type EMC3-FLAG or lacking EMC (ΔE). ApraA was included where indicated. The SPCs were recovered by centrifugation and analyzed by SDS-PAGE and autoradiography. Quantification of %glycosylation (indicative of insertion in the N_{exo} orientation) from three such experiments is shown at the bottom.

SUPPLEMENTAL REFERENCES

S1. Higy, M., Junne, T., and Spiess, M. (2004). Topogenesis of membrane proteins at the endoplasmic reticulum. Biochemistry *43*, 12716–12722. 10.1021/bi048368m.

S2. Chitwood, P.J., and Hegde, R.S. (2019). The Role of EMC during Membrane Protein Biogenesis. Trends Cell Biol. *29*, 371–384. 10.1016/j.tcb.2019.01.007.

S3. Chandrasekaran, V., Juszkiewicz, S., Choi, J., Puglisi, J.D., Brown, A., Shao, S., Ramakrishnan, V., and Hegde, R.S. (2019). Mechanism of ribosome stalling during translation of a poly(A) tail. Nat. Struct. Mol. Biol. *26*, 1132–1140. 10.1038/s41594-019-0331-x.

S4. Feng, Q., and Shao, S. (2018). In vitro reconstitution of translational arrest pathways. Methods *137*, 20–36. 10.1016/j.ymeth.2017.12.018.
